# Immune Modulation and Evasion by *Cryptococcus* spp.: An Integrative Review of Molecular Mechanisms and Experimental Evidence

**DOI:** 10.1021/acsomega.6c04202

**Published:** 2026-08-22

**Authors:** Yohana Porto Calegari-Alves, Luana Carolina Cardozo, Walter Orlando Beys-da-Silva, Lucélia Santi

**Affiliations:** † Programa de Pós-graduação em Biologia Celular e Molecular, 28124 Universidade Federal do Rio Grande do Sul, Porto Alegre, Rio Grande do Sul 90610-000, Brazil; ‡ Faculdade de Farmácia, Universidade Federal do Rio Grande do Sul, Porto Alegre, Rio Grande do Sul 90610-000, Brazil

## Abstract

The *Cryptococcus* species complex are major human pathogenic fungi capable of causing cryptococcosis, a severe pneumonia and meningitidis in both immunocompromised and, in some cases, immunocompetent patients. Their pathogenic success depends not only on classical virulence factors, but also on a coordinated capacity to evade, modulate, and exploit host immune responses across multiple stages of infection. In this review, we synthesize experimental evidence on the principal mechanisms that support cryptococcal immune evasion, integrating findings from molecular, cellular, and host–pathogen interaction studies. We discuss how surface remodeling and capsule organization reduce pathogen-associated molecular pattern exposure and impair immune recognition; how intracellular survival is promoted through phagosomal manipulation, altered trafficking, and nonlytic escape; and how dissemination is facilitated by phenotypic plasticity and flexible strategies for blood–brain barrier traversal. We further examine the contribution of metabolic adaptation, antioxidant defenses, and morphological heterogeneity to fungal persistence under host-imposed stress, as well as the ways in which *Cryptococcus* interferes with antigen presentation and protective immune polarization. By organizing these mechanisms within a unified framework, this review highlights how immune evasion in *Cryptococcus* is mediated not by a single determinant, but by an integrated and dynamic network of structural, metabolic, and immunoregulatory processes. A clearer understanding of these interconnected strategies may help refine mechanistic models of cryptococcal pathogenesis and inform future host-directed and antifungal therapeutic approaches.

## Graphical abstract

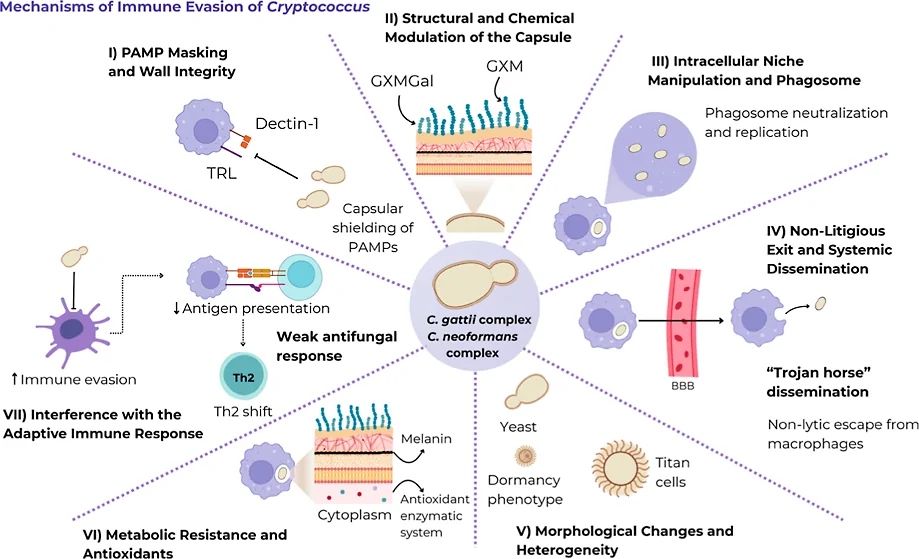

## 1 Introduction

Every year the number of people infected by fungal pathogens in the world increases alarmingly. One explanation for this increase is the growing number of individuals with immune system problems, such as HIV infection, transplants, prolonged immunosuppressive therapy, and neoplasms.^1,2^ Data published by the Global Fund for Fungal Infection Action estimates that approximately one billion people suffer from some type of fungal infection^3^ and more than 1.5 million die from complications caused by these infections.^4^ Addressing this public health challenge, the World Health Organization has included invasive mycoses in the neglected disease category. Specifically, the *Cryptococcus neoformans* and *Cryptococcus gattii* species complexes have been designated as critical and medium priority pathogens, respectively, to guide global research and surveillance efforts.^5^

Cryptococcosis is a systemic mycosis caused by encapsulated yeasts of the *C. neoformans* and *C. gattii* species complexes, which affect humans and other mammals, resulting in severe clinical manifestations such as pneumonia and, mainly, meningitis.^6^ *C. neoformans* is cosmopolitan and generally associated with environments contaminated by pigeon droppings, being responsible for infections in immunocompromised individuals, especially those with HIV/AIDS^7^ and it is considered the most lethal fungus in humans.^8^ In turn, *C. gattii* is more prevalent in tropical and subtropical regions, with an ecological association with trees of the genus *Eucalyptus* ^9^ and mainly affects immunocompetent people, being considered a primary pathogen.

Lung infections are generally milder due to the lack of a favorable environment for fungal growth and multiplication, which is facilitated by the efficient immune system’s goal of eliminating this pathogen. The first is the presence of alveolar macrophages, which line the alveoli and are responsible for phagocytizing and eliminating the microorganism. Additionally, some cells, such as T lymphocytes (CD4 and CD8) and dendritic cells (DCs), are recruited to the organ. Finally, the lung contains surfactants that, in addition to maintaining the surface tension of the pleura during respiration, have antimicrobial properties.^10^ Despite all these mechanisms, fungal cells can still survive in this environment and multiply inside macrophages,^11^ conferring advantages for the dissemination of the fungus.^12^ One example is the formation of cryptococcomas, which, although more common in *C. gattii*,^13^ has already been described in *C. neoformans* infections.^14–16^

However, even with all the human apparatus to eliminate the fungus, *Cryptococcus* has developed mechanisms to evade the human immune system that are still little understood. Among the mechanisms already characterized, the evasion of macrophages,^17^ DCs,^18^ neutrophils^19^ and T lymphocytes stand out.^20^ Other studies cite virulence factors, such as the polysaccharide capsule and melanin, which inhibit phagocytosis and minimize the enzymatic action generated by the immune response, respectively.^21,22^ Furthermore, the fungus has the ability to enter dormancy, becoming less detectable by immune cells,^23^ or to form titan cells, making them difficult for macrophages to eliminate due to their size.^24^

Given the global health burden of cryptococcosis and the intricate interplay between *Cryptococcus* spp. and the host immune system, a deeper understanding of the molecular mechanisms involved in immune evasion is paramount. While established virulence factors contributing to immune escape have been extensively documented, critical aspects of immune modulation remain poorly characterized, particularly in *C. gattii*. In this context, this review aims to synthesize the primary immune evasion mechanisms already described in the *C. neoformans* and *C. gattii* species complexes, while identifying conceptual gaps to guide future molecular investigations aimed to elucidate key targets of cryptococcal virulence.

## 2 Methodology

This review was conducted to synthesize the current state of knowledge on the mechanisms of immune system evasion by pathogenic fungi, from a molecular biology perspective. Bibliographic searches were conducted in May 2025 and updated in November 2025, in the PubMed, Scopus, and Web of Science databases. The search strategy was designed to encompass studies that used molecular biology to understand immune system evasion by *Cryptococcus*. The terms used were: (″immune evasion″ OR ″immune escape″ OR ″host–pathogen interaction″) AND (″*Cryptococcus*″ OR ″*C. neoformans*″ OR ″*C. gattii*″). Case reports, reviews, editorials, and brief communications without infection data, as well as studies focused exclusively on antifungal susceptibility assays without immune response analysis, were excluded. Titles and abstracts were analyzed to remove duplicates and identify studies that met the inclusion criteria (*n* = 268). After initial screening (*n* = 46), full texts from 2003 to 2025 were analyzed, and data extraction focused on the fungal species studied; the methodology and biological model used; and the main biological pathways or proteins/genes identified as crucial for immune evasion. The data were organized qualitatively, categorizing evasion mechanisms by type of immune response and by level of molecular regulation, allowing for an integrated discussion on how studies involving molecular biology are assisting in mapping fungal pathogenicity. The articles used in this review are presented in Table 1.

**Table 1:** Articles Selected for This Review

	Pathogen	Key factor	Evasion mechanism	Methodology	ref
PAMP masking and wall integrity	C. neoformans H99 and mutants (*rim101*Δ, *rim101*Δ RIM101, *cap59*Δ*rim101*Δ and *cap59*Δ)	Rim101	it promotes cell wall organization and efficient capsule attachment, resulting in the concealment of immunogenic epitopes and reduced detection by host phagocytic cells	transmission electron microscopy (TEM), Fluorescence microscopy, RNA-Seq, ELISA and Western blot. In vivo (A/J and C57BL/6 mice)	^25^
	C. neoformans *H99* and mutants *(rim101* Δ*:: NAT, rim101* Δ*::NAT + RIM101, chs3* Δ*:: NEO, chs3* Δ*:: NEO rim101* Δ*:: NAT, chs5* Δ*:: NEO, chs5* Δ*:: NEO rim101::NAT, cap59* Δ*:: NEO, cap59* Δ*:: NEO aro101* Δ*:: NAT)*	Rim101 pathway	the Rim101 pathway regulates cell wall structure by masking *N*-acetylglucosamine oligomers (chitin and chitosan) that could be recognized by the immune system	fluorescence Microscopy, Biochemical Analysis and MBTH Assay. In vivo model (BALB/c mice)	^26^
	C. neoformans H99 and mutants *fbp1*Δ and *fbp1*Δ*FBP1*	FBP1	involved in the proteasomal degradation of regulatory proteins, absence of FBP1 alters cellular homeostasis and cell wall composition	confocal microscopy and TEM, RT-qPCR, Western blot, ELISA and flow cytometry. In vivo model (A/Jcr and C57BL/6J mice)	^27^
	C. neoformans H99 and mutants	MAR1	it controls intracellular trafficking and localization of the β-(1.3)-glucan synthase Fks1, regulating the correct deposition of glucans and mannans in the cell wall	RT-PCR, Western blot, fluorescence and confocal microscopy, transmission electron microscopy (TEM), HPLC, enzyme assays, immune activation assays. In vivo model (C57BL/6 mice)	^28^
	C. neoformans H99 and mutant *ccr4*Δ	CCR4	responsible for degrading ribosomal protein transcripts, allowing for transcriptome and translatome reprogramming during heat stress, causing antigen masking	RNA-seq, Northern blot, qRT-PCR, Polysome profiling, Confocal microscopy and flow cytometry. In vivo model (Balb/cJ mice)	^29^
	C. neoformans H99 and mutant *ccr4*Δ	HOG-MAPK and CWI-MAPK pathways	translatome reprogramming and post-translational modulation of transcription factors cause β-1,3-glucan masking in response to thermal stress	Northern blot, RT-PCR, Western blot and Polysome profiling	^30^
	C. neoformans H99 and mutants (*csn1201*Δ and *csn1201*Δ*+CSN1201*)	CSN1201	promoting lung growth and extrapulmonary dissemination by modulating innate and adaptive host responses, thus increasing the pathogenic fitness of the fungus	Southern blot, phenotypic assays, transmission electron microscopy (TEM), and flow cytometry. In vivo model (Galleria mellonella and female Balb/C mice)	^31^
structural and chemical modulation of the capsule	C. neoformans H99, J4, J8, J20, 247, ATCC2406 and C. deneoformans B3501	capsule	spatial modulation of C3 deposition in the capsule, promoting intramatrix complement sequestration and reducing CR3-mediated recognition and phagocytosis, especially in strains with a large capsule	phatocytosis assays, confocal microscopy and measurement capsule	^32^
	C. neoformans H99	capsule	promotion of transient antigenic variations in the capsule during translocation across the BBB, hindering phagocytosis and favoring persistence in the CNS	immunohistochemistry, transmission electron microscopy (TEM), and morphometric analyses of the capsule. In vivo model (OF1 male mice)	^33^
	C. neoformans H99 and mutant *lhc1*Δ	LHC1	remodeling of the tertiary structure of the polysaccharide capsule, modulating its permeability, electrical charge, and interaction with the complement system	enzyme assays, measurement of capsular size, and RT-qPCR	^34^
	C. neoformans RC-2	ALL1	inactivation of the ALL1 gene causes the fungus to have a larger capsule, escape phagocytosis and cause more severe infections, increasing virulence and favoring fungal persistence	microarray, RT-PCR, optical and confocal microscopy, ELISA, immunohistochemistry and phenotypic characterization. In vivo model (BALB/c mice)	^35^
	C. neoformans H99 and C. deuterogattii (JP02 - Japan isolated)	GXM	alterations in the structure of GXM, with loss of O-acetyl, reduce the activation of dendritic cells	flow cytometry, ELISA and RMN. In vivo model (Balb/cJ mice)	^36^
	C. deuterogattii R265 and mutants (*krp1* **Δ** and *krp1* Δ*:: KRP1*)	KRP1	maintaining capsular integrity and a balance between polysaccharide retention and secretion, influencing the initial interaction with the immune system	confocal microscopy, GC–MS, physicochemical characterization of GXM, ELISA, Western blot, phenotypic analyses, phagocytosis assay, and flow cytometry. In vivo model (BALB/c mice)	^37^
	C. neoformans H99 and B3501	biofilm and capsule	biofilm formation results in cells with enlarged capsules, increased release of capsular polysaccharides, and modifications to the cell surface, hindering phagocytosis and elimination by macrophages	metabolic activity assay, flow cytometry and microscopic phagocytosis assay, ELISA, qRT-PCR and immunofluorescence	^38^
	C. deuterogattii PNG18 and mutants (Δ*CAP10,* Δ*CAP59,* Δ*CAP60* and Δ*CAP64)*	capsular polysaccharides	extracellular CPs can coat capsule-deficient strains, being sufficient to prevent fungal recognition mediated by CD11b	scanning electron microscopy (SEM) and confocal microscopy, ELISA, Western blot, and enzyme assays. In vivo model (C57BL/6J mice)	^39^
	C. neoformans H99 and mutants *tps1*Δ and *tps1*Δ*/TPS1*	TPS1	it evades resident pulmonary and innate defense mechanisms, likely by facilitating the formation of the cryptococcal capsule	enzyme assays and capsule quantification. In vivo model (Balb/c mice)	^40^
	C. neoformans H99 and mutants Δ*cgm1,* Δ*cgm2,* Δ*cap59* and Δ*cgm1*Δ*cgm2*	CGM1	promoting the correct assembly of GXMGal, contributing to a functional capsule that reduces the fungicidal activation of macrophages	enzyme assays, NMR and in silico assays. In vivo model (C57BL/6 mice)	^41^
intracellular niche manipulation and phagosome	C. neoformans H99, ATCC 24067, CAP67 and B3501	intracellular replication	release of PCs within human monocytes causes inhibition of phagosome-lysosome fusion, resisting oxidative stress and exit by oxidative exocytosis	transmission electron microscopy (TEM), confocal fluorescence microscopy, Immunohistochemistry and Histopathological analysis. In vivo model (C57BL/6, A/JCr and 129/SvEv mice)	^42^
	C. neoformans H99 and mutants (*cap59* e *xl1601*)	ATG proteins (ATG2A, ATG5 and ATG9A)	subversion of the host’s autophagy pathway	fluorescence microscopy, immunofluorescence assay, and Western blotting	^43^
	C. neoformans H99 and mutants (*ure1*Δ*, ure7*Δ*, ure7*Δ*/URE7, plb1*Δ*, plb1*Δ*/PLB1, sec14–1*Δ*and sec14–1*Δ*/SEC14–1*) and strain B3501 and mutant *cap67*Δ	RAB5 and RAB11	modification of the phagolysosome maturation process, inducing premature removal of initial phagosome markers, as well as causing significant acidification that impairs protease activity	high-resolution confocal microscopy, Transmission electron microscopy (TEM), Immunofluorescence, and Western blot	^44^
	C. deuterogattii R265, R272 and WM276	F-actin	formation of a ″cage″-like structure around the phagosome that prevents binding with the lysosome and consequently activation of the immune response	immunofluorescence microscopy and flow cytometry	^45^
	C. neoformans cepa H99 and mutants (*ure1*Δ and *ure1*Δ*::URE1*)	urease	it increases the pH of the phagolysosome, interfering with the maturation and intracellular replication of the fungus, the balance between replication, vomitocytosis and damage to the phagosome, and influencing brain invasion by macrophages (″Trojan horse″)	confocal microscopy, flow cytometry, phagolysosomal pH measurement. In vivo model (C57BL/6 mice)	^46^
	C. deuterogattii R265 and mutants (*cap10*Δ, *cap59*Δ and *cap59R*)	capsule	masking of extracellular detection and reduction of phagocytosis	Southern and Western blot, measurement of capsular size, enzymatic assays, and confocal microscopy	^47^
	C. neoformans H99 and mutants Δ*plb1* and Δ*plb1::PLB1*	PLB1	permeabilization of the phagolysosomal membrane	qRT-PCR, Western blot, flow cytometry, and confocal and fluorescence microscopy	^48^
non-litigious exit and systemic dissemination	C. neoformans H99	nonlytic exocytosis	persistence within monocytes and escape via nonlytic exocytosis	confocal fluorescence microscopy and flow cytometry	^49^
	C. neoformans H99, Jec21 and B3501			confocal fluorescence microscopy and flow cytometry	^50^
	C. neoformans H99 and B3501 and mutants *CAP67*			flow cytometry and microscopy. *In vivo* model (BALB/c mice)	^51^
	C. neoformans H99 and 24,067			Transmission electron microscopy (TEM)	^52^
	C. neoformans H99 and mutants (*ure1*Δ and *cps1*Δ)	“Trojan horse”	use of phagocytes as vehicles to cross the blood–brain barrier	confocal microscopy, transmission electron microscopy (TEM), and enzyme assays	^53^
	C. neoformans H99,BT63, JEC20, JEC21, B3502 and B3501	spore	exploitation of pulmonary phagocytes as vehicles for systemic dissemination (“Trojan Horse”)	fluorescence microscopy and flow cytometry. In vivo model (female C57Bl/6 and CD11c-DTR mice)	^54^
	C. neoformans H99 and mutants (Δ*SOD1*, Δ*CAT1* and Δ*GPx1*)	vomocytosis and dragocytosis	persistence within monocytes and escape via nonlytic exocytosis and/or macrophage-to-macrophage transfer	confocal microscopy, measurement of capsular size, and enzymatic assays	^55^
morphological changes and heterogeneity	C. neoformans RC-2	senescence process	microevolutionary mechanisms that generate capsular enlargement, hypervirulence, and *in vivo* accumulation	optical and fluorescence microscopy, phenotypic assays, and enzymatic assays. *In vivo* model (BALB/c mice)	^56^
	C. neoformans KN99α and mutants (*cap67* and *cap59*)	titan cells	formation of cells with thicker cell walls and denser, highly cross-linked capsules	electron microscopy and phenotypic assays	^57^
	C. neoformans H99 and human clinical isolates	dormancy phenotype	decreased overall metabolism with low growth leading to a stage of latency and persistence	flow cytometry, contrast microscopy and RT-qPCR. *In vivo* model (OF1 e BALB/c mice)	^58^
metabolic resistance and antioxidants	C. neoformans 2E-TU-4 and 2E-TUC-4	CNLAC1	melanin production aids in the survival of fungi in pulmonary macrophages due to pH alteration	Elisa and Histopathology assays. *In vivo* model (Female CBA/J mice)	^59^
	C. neoformans H99 and mutants (*gpx1*Δ and *gpx2*Δ)	Gpx1 and Gpx2	neutralization of reactive oxygen species	Southern blot, RT-qPCR, Fluorescence microscopy and Enzyme assays	^60^
	C. neoformans H99 and mutants (Δ*irk2* and Δ*irk5*)	Irk2 and Irk5	modulation of mitochondrial metabolism pathways and ATP production, affecting fungal survival in the face of oxidative and thermal stress during infection	quantitative proteomics by LC–MS/MS, nontargeted metabolomics, and enzymatic assays	^61^
	C. neoformans H99 and mutants (Δ*clpX* and Δ*clpX::CLPX*)	CLPX	it acts in mitochondrial protein quality control, participating in heme synthesis, iron homeostasis, and maintenance of the ergosterol pathway	quantitative proteomics by LC–MS/MS, RT-qPCR, phenotic and enzymatic assays. *In vivo* model (C57BL/6 mice)	^62^
interference in the adaptive response	C. neoformans H99 and mutants *(plb1 and plb1rec)*	PLB1	production of cryptococcal eicosanoids that negatively modulate the activation of macrophages and neutrophils	histopathological analysis, ELISA and fluorescence microscopy. *In vivo* model (CBA/J mice)	^63^
	C. neoformans isolated from patient	Virulence-associated DEAD-box RNA helicase (VAD1)	modulation of the cytokine profile, promoting a Th2 response in the acute phase of infection, which is less efficient in eliminating pathogens, causing an imbalance between Th1 and Th2 responses	RT-qPCR, ELISA, and enzyme assays	^64^
	C. neoformans and mutants *(cap59D, cas1D and uge1D)*	NF-κB (transcription factor)	reduction of macrophage proliferation (capsule-independent) and induction of macrophage apoptosis (capsule-dependent)	Flow cytometry, Western blot, and immunohistochemistry. *In vivo* model (Transgenic κB-lacZ mice)	^65^
	C. neoformans H99 and mutants *(KN99α, uxt1*Δ*, UXT1, uxt2*Δ*, UXT2, uxt1*Δ*uxt2*Δ*, uxs1*Δ *and cap59*Δ*)*	Xylose	promoting an ineffective Th2 response and hindering the formation of organized immune structures	ELISA, histology and immunofluorescence. *In vivo* model (C57BL/6J and a/jcR mice)	^66^
	C. neoformans H99	capsule	release and accumulation of GXM in the CNS, silencing inflammation and dysfunction in the local response	histopathology, morphology, immunohistochemistry and confocal microscopy. *In vivo* model (C57BL/6 female mice)	^67^
	*C. deneoformans* B3501	CARD9	modulation of the adaptive immune response due to interference in the induction and activation of resident memory T cells in lung tissue	flow cytometry and histochemical analyses. *In vivo* model (C57BL/6 female mice)	^68^
	C. gattii R265, R272, WM276 and clinical strains	hypervirulent strains	targeted suppression of the macrophage response, blocking the M1/Th1 microbicidal profile and favoring a permissive M2/Th2 state, weakens antigen presentation and oxidative stress	ELISA, RT-qPCR, enzyme assays and dual RNA-seq. *In vivo* model (C57BL/6 female mice)	^69^

### 2.1 PAMP Masking and Cell Wall Integrity

The success of infection depends on the ability to transition from the natural environment to the hostile environment of the mammalian host, a transition that requires, among many factors, alterations in its surface architecture. The fungal cell wall is the first point of contact between *Cryptococcus* and the cells of the innate immune system, serving as a critical interface for the recognition of Pathogen-Associated Molecular Patterns (PAMPs), a dynamic process where the fungus conceals its most immunogenic structural components, such as β-glucans and chitin, under a complex polysaccharide capsule and a meticulously organized cell wall.^56^

The surface of *Cryptococcus* is distinct among human fungal pathogens due to the presence of a polysaccharide capsule, composed primarily of glucuronoxylomannan (GXM) and galactoxylomannan (GalXM). The capsule functions as the main virulence determinant, physically blocking access by host pattern recognition receptors (PRRs) to cell wall polysaccharides. However, the effectiveness of this masking depends entirely on the structural integrity of the underlying cell wall, which is composed of a lamellar network of α-(1,3)-glucan, β-(1,3)-glucan, β-(1,6)-glucan, chitin, chitosan, and mannoproteins. Unlike *Candida albicans*, where β-glucan is the innermost layer, in *Cryptococcus* the lamellar organization and the presence of chitosan in high proportions, up to 10 times greater than chitin in some phases,^70^ confer a unique plasticity since its absence results in attenuated virulence and rapid elimination by the host.^71^

In addition to the overall architecture of the cryptococcal cell wall, the equilibrium between chitin and chitosan is itself a major determinant of immune masking. In *Cryptococcus*, chitin is extensively deacetylated by the chitin deacetylases CDA1, CDA2 and CDA3, generating chitosan, a polymer that contributes to wall flexibility, structural integrity, and reduced immune detectability.^72,73^ This modification is not only biochemical, thereby supporting persistence and virulence.^74^ The importance of this process is underscored by the fact that loss of chitosan homeostasis destabilizes the wall and favors immune clearance. This framework is especially relevant in titan cells, which display increased glucosamine content consistent with enhanced chitin/chitosan deposition and major remodeling of the wall during infection.^57^

One of the crucial discoveries in understanding antigen masking in *Cryptococcus* is the role of the Rim101 transcription factor.^25^ This pathway is conserved in fungi for pH response, but in *Cryptococcus*, it has been adapted to specifically regulate the host–pathogen interface. When the fungus enters the host and encounters the physiological alkaline pH, the Rim101 pathway is activated, resulting in proteolytic cleavage of Rim101 into its active form, which then translocates to the nucleus to orchestrate cell wall gene transcription and capsule synthesis. The absence of *Rim101* gene results in a catastrophic failure in cell wall organization, despite the fungus being able to synthesize and secrete normal levels of capsular polysaccharide.^26^ The phenotype of the *rim101*Δ*m*utant under capsule-inducing conditions is characterized by an extremely thick and disorganized cell wall, which prevents proper capsule anchoring, leaving the wall exposed.^26^ In lung infection models, the *rim101*Δ*m*utant induces a hyperinflammatory response characterized by a massive influx of neutrophils and eosinophils into the lung, elevated levels of pro-inflammatory cytokines, and paradoxical hypervirulence.^26^

While *Rim101* acts as a global transcriptional regulator, the MAR1 protein (Macrophage Activating Regulator of cell wall-1) has also been identified as an essential component for the intracellular trafficking of cell wall synthases. MAR1 is a membrane protein with conserved transmembrane domains that dynamically localizes in response to environmental conditions.^28^ Cell wall integrity and consequent antigen masking depend on the coordinated transport of polysaccharide-synthesizing enzymes to the plasma membrane. In the *mar1*Δ*m*utant, a specific defect in β-glucan synthase (*Fks1* gene) trafficking is observed.^28^ Although the MAR1 protein has been linked to FKS1 trafficking, the molecular details of how vesicles transporting polysaccharides and synthases navigate the cryptococcal cytoskeleton in response to stress remain unclear. The identification of specific motor and adaptor proteins that interact with *Mar1* and *Rim101* is still unknown. However, while in wild-type cells exposed to stress conditions Fks1-GFP is enriched in the plasma membrane, in the *mar1*Δ*m*utant, the enzyme remains sequestered in internal compartments of the cytoplasm.^28^ This trafficking failure has direct consequences on the composition of the outer layer of the cell wall. Furthermore, a significant reduction in glucans and mannans was observed in the glucose and mannose levels in the cell wall of the *mar1*Δ*m*utant, resulting in the exposure of chito-oligomers and a defect in capsid anchoring.^28^

Recently, the CSN1201 protein was identified as a novel virulence determinant in *C. neoformans*.^31^ This protein contains a PCI domain (Proteasome, COP9, Initiation factor 3), suggesting a role in stabilizing protein–protein interactions within multiprotein complexes. Interestingly, *Csn1201* appears to be evolutionarily specific to certain fungal species, being absent in *Saccharomyces cerevisiae* and humans. Deletion of the *Csn1201* gene results in profound alterations in cell surface architecture when the fungus is exposed to the host, such as increased cell wall thickness, reduced capsule size, and exposure of PAMPs. Unlike other mutants, *csn1201*Δ*i*nduces a protective immune response in the host. In inhalation models, preferential accumulation of DCs in the lungs and increased polarization toward Th1 and Th17 responses were observed.^31^ Furthermore, the mutant exhibits reduced intracellular survival in macrophages and a diminished ability to cross the blood–brain barrier (BBB).^31^

Antigen masking in *Cryptococcus* is not only a response to pH or nutrient availability, but is also related to thermotolerance and the fungus’s ability to reprogram its translatome in response to body temperature (37 °C). The CCR4 protein, the main deadenylase of the Ccr4-Not complex, is the central piece of this mechanism.^29^ In *C. neoformans*, entry into the host triggers rapid, Ccr4-mediated degradation of mRNAs encoding ribosomal proteins. This degradation is essential as it frees up ribosomes to translate stress response mRNAs and transcription factors. Furthermore, it contributes to the masking of PAMPs, as observed in the *ccr4*Δ*m*utant, which exhibited anomalous unmasking of β-(1.3)-glucan specifically at 37 °C, leading to direct recognition by Dectin-1 in pulmonary macrophages. As a consequence, a significant increase in phagocytosis was observed, with a reduction in the fungus’s ability to evade innate immune surveillance. The relationship between the failure to degrade ribosomal mRNAs and the repression of masking genes in the *ccr4*Δ*m*utant still requires definitive mechanistic proof that competition for ribosomes is the sole factor.

Masking regulation also involves mitogen-activated protein kinase (MAPK) cascades. The main HOG (High-Osmolarity Glycerol) and CWI (Cell Wall Integrity) pathways of β-(1.3)-glucan in *C. neoformans*.^30^ The first is composed of Ssk2, Pbs2, and Hog1, and mutants in this pathway showed moderate unmasking at 37 °C compared to wild-type. The second is led by the Mpk1 kinase (Slt2), and mutants in this pathway exhibit extensive unmasking and, unlike the HOG pathway, does not impact translatome reprogramming, suggesting that it regulates masking through post-translational modifications of transcription factors or structural proteins already present in the cell wall.

Antigen masking and fungal immunogenicity are also modulated by controlled protein turnover. The F-box protein (FBP1), part of the SCFFbp1 E3 ligase complex, has been identified as a virulence determinant that shapes the immunogenic potential of the fungus.^27^ The *fbp1*Δ*m*utant is hypovirulent and incapable of causing meningitis, despite maintaining normal capsule production, melanin, and thermotolerance. The attenuation mechanism is linked to the induction of a robust immune response of monocyte recruitment and Th1/Th17 polarization. This suggests that FBP1 normally functions to suppress fungal immunogenicity, possibly by degrading cell wall proteins that would act as early warning signals to the immune system.

In conclusion, antigen masking in *Cryptococcus* is a complex and multidimensional survival strategy that functions via a dense regulatory network that goes beyond the capsule’s physical barrier (Figure 1). Fungal surface integrity is actively adjusted in response to host pressures, as evidenced by the cooperation of transcription factors like Rim101, MAR1-mediated vesicular trafficking, protein stability via CSN1201 and FBP1, and translatome reprogramming by CCR4. In addition to elucidating the mechanisms of fungal pathogenesis, an understanding of how these pathways converge to conceal immunogenic ligands reveals molecular vulnerabilities that may be exploited to develop novel therapeutic strategies.

**Figure 1:**
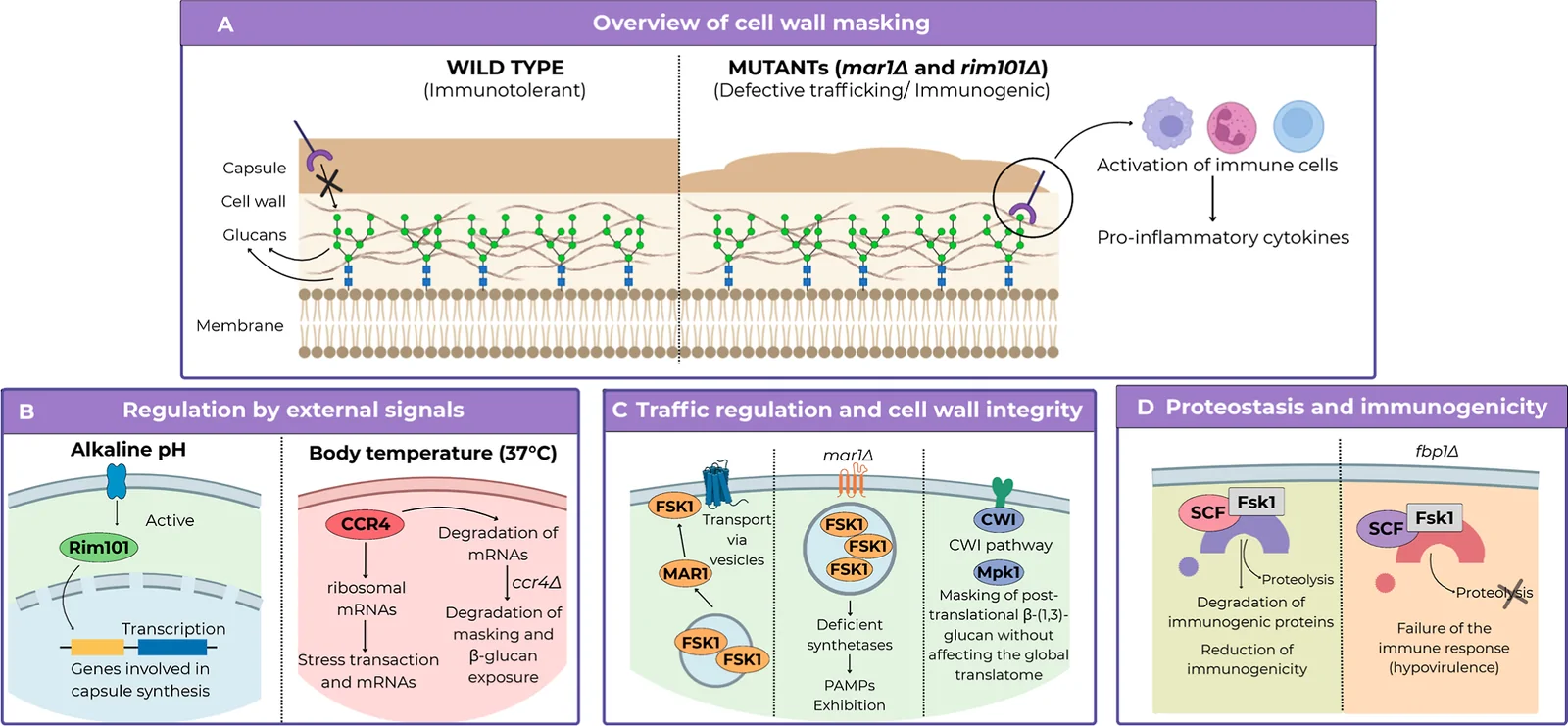
Overview of the regulatory mechanisms that control fungal cell wall masking and immune recognition. (A) Comparison of wild-type (immunotolerant) and mutant (immunogenic) cell surfaces. An intact capsule masks pathogen-associated molecular patterns (PAMPs), preventing immune recognition. Defective masking in mutants leads to PAMP exposure, activating immune cells; (B) environmental signals (pH, temperature) regulate cell wall synthesis (via Rim101) and stress translation (via CCR4). Disruption leads to defective masking; (C) vesicular trafficking (mediated by MAR1) and the cell wall integrity signaling pathway (mediated by Mpk1) integrate glucan synthetase transport and β-glucan masking. Defects result in internalized vesicles and PAMP exposure; (D) the SCF̂(Fbp1) complex mediates proteolysis of immunogenic cell wall proteins (e.g., Fks1), reducing fungal immunogenicity and maintaining virulence. Failure of this proteolysis increases immunogenicity, leading to hypovirulence. Illustration created by the authors using Biorender (https://www.biorender.com/) and Canva (https://www.canva.com/). Graphical elements were used under valid publication licenses in accordance with the respective platform license agreements. Therefore, no additional permission from third-party copyright holders was required.

### 2.2 Structural and Chemical Modulation of the Capsule

The polysaccharide capsule of fungi of the genus *Cryptococcus* is the major determinant of virulence and a sophisticated tool for immune evasion.^19^ Modulation of the macromolecules that compose it, both in terms of their primary chemical structure and their higher-order organization, enables the fungus to conceal antigenic epitopes, inhibit opsonization, and manipulate cytokine signaling in host immune cells.^75^ Over the last decades, the understanding of cryptococcal virulence has progressed from structural descriptions of capsular polysaccharides to investigations of how these molecules are organized and processed after secretion. A milestone in this field was the identification of lactonohydrolase 1 (LHC1), a secreted, capsule-associated enzyme that regulates GXM processing.^34^ Molecular-driven approaches focused on capsular proteins showed that LHC1 acts as a critical determinant of capsule density and branching. In the *lhc1*Δ*m*utant, the capsule is significantly larger but structurally disorganized.^34^

Biophysical approaches further demonstrated that, in wild-type strains, LHC1 reduces capsule dimensions and alters its structural complexity.^34^ In contrast, *lhc1*Δ*e*xhibits GXM polymers with increased average molecular mass and hydrodynamic radius, consistent with defective enzymatic processing of newly exported units directly impacting complement interactions. While the compact wild-type capsule restricts C3 deposition, the more porous *lhc1*Δ*m*atrix promotes increased and heterogeneous deposition, culminating in nearly 2-fold higher phagocytosis by J774.16 macrophages. Thus, LHC1 contributes to virulence by compacting the capsular shield and reducing its permeability to innate defenses.

Beyond limiting opsonization, the cryptococcal capsule impairs complement biology by sequestering C3 fragments within its dense matrix. This spatial reorganization prevents the efficient presentation of opsonins to phagocyte receptors like CR3 at the fungus-host interface.^76^ Consequently, complement activation is dissociated from fungal elimination, as embedded fragments remain functionally inaccessible, rendering phagocytosis inefficient despite apparent labeling.

Furthermore dictating complement deposition patterns, the capsular polysaccharides themselves act as direct ligands that reprogram phagocyte effector function once opsonic barriers have already been negotiated. GXM impairs Fcγ receptor and complement receptor-mediated internalization of the yeast, and even when partial uptake occurs, it interferes with the intracellular signaling required for phagosome-lysosome fusion.^77^ In canine and murine macrophage models, exposure to purified capsular polysaccharide markedly suppresses nitric oxide synthesis and reduces proteasomal activity, two effector arms that converge on intracellular fungal killing, indicating that the capsule undermines microbicidal capacity independently of its role in blocking initial recognition.^78^ This functional suppression is compounded by a cytokine profile that favors fungal persistence: GXM dampens classical pro-inflammatory mediators and attenuates NF-κB signaling in phagocytes, reinforcing, at the transcriptional level, the same anti-inflammatory bias achieved structurally through complement sequestration and opsonin masking.^77^

This structure also helps explain why the capsule’s architecture, and not just its size, is an important determinant of immune evasion. A compact and highly organized matrix restricts penetration, redistributes opsonic molecules, and limits productive receptor engagement, whereas a more porous capsule increases complement accessibility and favors fungal uptake by phagocytes.^18^ Thus, complement evasion by the fungus should be viewed as a spatial immunobiology problem, since the fungus not only avoids complement but also reorganizes how it interacts with the capsular network in ways that dissociate deposition from elimination.

The consequences of capsular exposure for phagocyte biology extend beyond impaired microbicidal function to the induction of programmed cell death. Purified GXM and GXMGal both trigger apoptosis in alveolar and peritoneal macrophages through upregulation of Fas ligand (FasL/CD95L) on the phagocyte surface itself, engaging the extrinsic apoptotic cascade via caspase-8 cleavage.^79^ This receptor-mediated route is mechanistically distinct from the caspase activation that follows phagolysosomal membrane permeabilization described elsewhere in this review, representing an additional, capsule-intrinsic pathway by which *Cryptococcus* depletes the effector pool at the primary infectious focus, emptying the alveolar inflammatory infiltrate and sustaining a permissive local environment.^79^

Although GXM dominates capsular architecture, GXMGal plays an important qualitative role in pathogenesis, particularly by influencing adaptive immune responses. GXMGal is smaller than GXM and contains an α-galactan core decorated with complex galactomannan side chains modified with xylose and glucuronic acid.^41^ Deletion of CGM1, a GT139 family β-galactoside α-(1→4)-mannosyltransferase, leads to near-complete loss of mannose in the galactomannan side chain, resulting in truncated GXMGal. This change is associated with increased interferon-γ levels in the lungs of infected mice, suggesting that GXMGal integrity is required to dampen pro-inflammatory polarization, including activation of M1-type macrophages.^41^ In parallel, the *cgm1* mutant exhibits thermal sensitivity at 37 °C and reduced virulence, reinforcing the relevance of this modulation for survival under host stress. In wild-type strains, GXMGal appears to function as an immune “buffer”, limiting the onset of a robust Th1 response. The complexity of capsule biosynthesis is further evidenced by the need for multiple genes, such as *Ugt1* (UDP-Gal transporter) and *Uge1* (UDP-Gal 4-epimerase), for the functional assembly of GXMGal, where mutations in these genes result in attenuated virulence phenotypes and increased phagocytosis.

Notably, GXMGal’s role as an immune buffer is not one of uniform suppression but of context-dependent dual action. While its structural integrity dampens excessive IFN-γ-driven Th1/M1 polarization, purified GXMGal paradoxically induces robust TNF-α and nitric oxide production in peritoneal macrophages and in the RAW 264.7 lineage, revealing an intrinsic capacity to engage inflammatory pathways under specific stimulation conditions.^79^ A similar dichotomy is observed in dendritic cells, where GXMGal behaves as a potent activator rather than a purely inhibitory ligand, driving partial phenotypic maturation through modulation of costimulatory molecules.^80^ This altered maturation state redirects, rather than abolishes, antigen presentation, biasing subsequent T-cell priming toward a Th17 profile: dendritic cells conditioned with GXMGal and cocultured with T lymphocytes elicit strong IL-17 and IL-6 production.^80^ The IL-17 axis is functionally double-edged, supporting early neutrophil recruitment and pulmonary fungal containment while also carrying the potential, if left unchecked, to drive immunopathological tissue damage, underscoring that GXMGal’s immunomodulatory signature cannot be reduced to simple suppression or simple activation.^80^

Beyond architectural control, subtle chemical modifications of GXM profoundly modulate immunogenicity. A prominent example is the addition of O-acetyl groups to mannose residues in the GXM backbone, which represents a fine mechanism for regulating fungal immunogenicity. ^1^H NMR analyses revealed that discrete differences in acetylation patterns distinguish conventional strains from highly virulent isolates.^36^ The hypervirulent *C. gattii* strain JP02, for instance, displays an O-acetylation profile distinct from that of *C. neoformans* H99, lacking one of the two acetyl groups detected in the latter and generating consequences in the activation of DCs.^22^ The GXM derived from H99 induces IL-6, IL-12p40 and TNF-α; JP02-derived GXM behaves in an immunologically “silent” manner.^36^ The functional importance of O-acetylation was confirmed by chemical deacetylation, which abolished the capacity of capsular fractions from both species to induce cytokine release by DCs.^36^ The working hypothesis is that DCs do not directly recognize the acetyl group per se, but rather sense acetylation-induced conformational changes in GXM. From a pathogenic standpoint, such “chemical silencing” favors early evasion and may contribute to dissemination even in immunocompetent hosts by compromising the timely establishment of a protective Th1 response.

As noted above, the capsule also functions as a physical barrier, concealing cell–wall PAMPs and limiting engagement of pattern-recognition receptors. A central mechanism involves disruption of interactions between CD11b (integrin α_M) and fungal ligands.^39^ In acapsular mutants such as *cap59*Δ or *cap64*Δ, DCs and macrophages readily recognize protein antigens in the cell wall, activate SYK, and trigger phagocytosis and cytokine production. Reconstitution assays demonstrated that simply treating acapsular cells with extracellular polysaccharides restores immune evasion, even in the absence of a visible capsular layer by India ink staining.^39^ This polysaccharide coating masks protein ligands recognized by CD11b and reduces phosphorylation of components of the signaling cascade in DCs. Notably, this effect appears to be receptor-specific, as CD11b is blocked and receptors such as Dectin-1 can maintain variable interaction with the cell wall depending on culture conditions and structural remodeling.^39^ This model reinforces that secretion of GXM into the extracellular milieu is not metabolic waste, but an active “coating” strategy that prevents PRR recognition.

The receptor landscape engaged by capsular polysaccharides extends beyond surface lectins and integrins to endosomal sensors. Beyond the canonical TLR2, TLR4, Dectin-1, and DC-SIGN axis, Toll-like receptor 9 (TLR9) has emerged as a critical, if less appreciated, node in the systemic coordination of the anticryptococcal response. Loss of TLR9 signaling does not merely blunt local immune activation but translates into overt organ pathology, as demonstrated in murine models of systemic *C. gattii* infection, where TLR9 deficiency exacerbates ocular impairment and drives significant visual loss.^81^ This finding indicates that the net outcome of capsular ligand engagement, whether it resolves toward generalized immunosuppression or toward a compartmentalized, protective inflammatory response, depends on the coordinated integration of signals across both surface and endosomal pattern-recognition receptors, and not on any single receptor–ligand pair in isolation.^81^

Capsular polysaccharides also exert a bimodal control over neutrophil effector function, specifically over the release of neutrophil extracellular traps (NETs), the chromatin-and-granule-protein lattices that constitute a central strategy for extracellular fungal containment. GXM significantly inhibits both spontaneous and induced NET release by human neutrophils, whereas GXMGal acts in the opposite direction, actively stimulating NET extrusion.^82^ This functional divergence between the two major capsular constituents illustrates that the relative balance of GXM and GXMGal shed into the local microenvironment, rather than the sheer quantity of polysaccharide released, dictates whether infiltrating granulocytes are held in check or licensed to deploy an extracellular antifungal response.^82^

Capsular plasticity also accompanies changes in fungal lifestyle, including biofilm formation. *C. neoformans* cells derived from biofilms exhibit larger capsules and release substantially higher amounts of GXM into the supernatant than planktonic cells.^83^ Another form of variation observed in vivo is an epigenetic transition to a mucoid phenotype. The *All1* gene (Allergen 1), encoding a cytoplasmic protein, acts as a negative regulator of this state.^40^ Hypervirulent mucoid variants frequently show downregulation of ALL1, and *all1*Δ*m*utants recapitulate larger basal capsules but defects in capsule induction in response to external stimuli. Loss of ALL1 is associated with strong inhibition of phagocytosis and an ineffective, disorganized inflammatory response in lungs and brain, with massive yeast accumulation and increased intracranial pressure.^83^

Assembly of a robust and functional capsule is energetically costly and requires integrity of central metabolic pathways. The trehalose pathway, coordinated by trehalose-6-phosphate synthase (TPS1), has emerged as an essential pillar for efficient encapsulation.^40^ Although trehalose is not a structural capsule component, the *tps1*Δ*m*utant fails to reach typical capsule size either in vitro or in host lungs, exhibiting an atrophic capsule reminiscent of an acapsular phenotype. The immunological consequence of this metabolic failure is extremely rapid pulmonary clearance, occurring independently of CD4+ T cells.^40^ Without a fully formed capsule, the fungus becomes vulnerable to resident defenses and innate immunity, failing to colonize the CNS. These findings support that structural capsular modulation is coupled to trehalose-6-phosphate-mediated metabolic signaling; when this axis fails, the fungus cannot build its polysaccharide shield and loses virulence.

Finally, changes in capsular composition also affect colloidal properties and physical interactions with immune cells. The protein KRP1 contributes to the organization of cell–wall glucans, which serve as a platform for proper anchoring of polysaccharide fibers.^37^ The *krp1*Δ*m*utants display thicker capsular fibers and increased release of soluble GXM into the environment, which may intensify the systemic immunosuppressive effects of the polysaccharide.^84^

In summary, the *Cryptococcus* polysaccharide capsule is a dynamic, metabolically integrated organelle that is crucial for host adaptation rather than just a static physical barrier, and one whose reach extends well beyond passive shielding into the active reprogramming of innate and adaptive effector responses (Figure 2). As mentioned above, the success of this pathogen relies on a sophisticated hierarchy of control, ranging from the enzymatic processing of GXM by LHC1 and the biosynthetic branching of GXMGal by CGM1 to the chemical “silencing” mediated by O-acetylation patterns, layered atop direct immunomodulatory actions that suppress phagocyte microbicidal function, trigger Fas-mediated apoptosis, exert bimodal control over neutrophil NET release, and differentially steer dendritic cell maturation and Th17 polarization through GXMGal. The integration of these structural and immunomodulatory features with central metabolic signaling, such as the trehalose pathway, with endosomal pattern-recognition via TLR9, and with the spatial reorganization of complement components, underscores a complex strategy of immune evasion that dissociates pathogen recognition from effective elimination. Ultimately, deconstructing the interplay between capsular architecture, protein-mediated anchoring (via KRP1 and ALL1), and the active masking of cell–wall PAMPs and functional paralysis of phagocytes and granulocytes reveals that the cryptococcal surface is a programmable interface. Understanding these molecular underpinnings not only clarifies the mechanisms of fungal hypervirulence and dissemination to the CNS but also highlights potential therapeutic targets aimed at stripping the fungus of its protective coating and restoring host immune competence and dissemination to the CNS but also highlights potential therapeutic targets aimed at stripping the fungus of its protective coating and restoring host immune competence.

**Figure 2:**
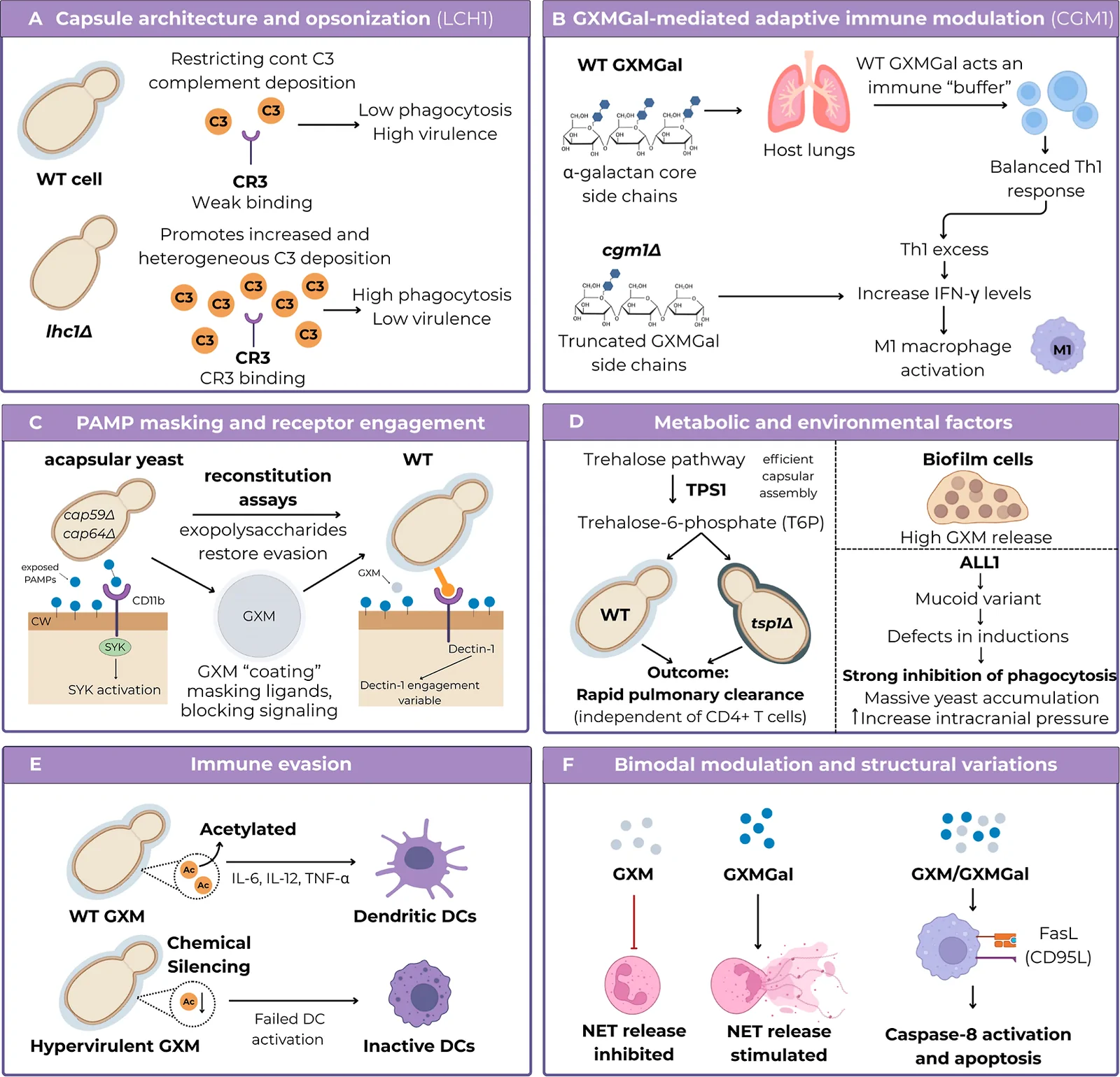
Structural and functional mechanisms by which the *Cryptococcus* capsule promotes immune evasion and host adaptation. (A) LHC1-dependent capsule remodeling regulates complement deposition and CR3-mediated phagocytosis. (B) GXMGal modulates adaptive immunity by limiting excessive Th1 polarization, meanwhile cgm1Δ enhances IFN-γ production and M1 macrophage activation. (C) Capsular polysaccharides mask PAMPs, reducing immune recognition. (D) Metabolic and environmental factors, including TPS1, biofilm formation, and ALL1, regulate capsule assembly, GXM release, and fungal virulence. (E) O-acetylation of GXM modulates dendritic-cell activation and inflammatory cytokine production. (F) GXM and GXMGal differentially regulate neutrophil extracellular trap (NET) formation and FasL-mediated macrophage apoptosis. Illustration created by the authors using Biorender (https://www.biorender.com/) and Canva (https://www.canva.com/). Graphical elements were used under valid publication licenses in accordance with the respective platform license agreements. Therefore, no additional permission from third-party copyright holders was required.

### 2.3 Manipulation of the Intracellular Niche and the Phagosome

After inhalation of environmental propagules, the first encounter with the immune system occurs in the pulmonary alveoli, where alveolar macrophages and DCs attempt to internalize and eliminate the invader.^85^ However, instead of being degraded, *Cryptococcus* has evolved a broad repertoire of mechanisms to subvert the intracellular niche, converting the phagosome into a permissive environment for replication, persistence, and eventual systemic dissemination.^44^ In murine models of pulmonary infection analyzed by electron microscopy, alveolar macrophages were observed to rapidly internalize yeasts following intratracheal exposure and to remain associated with the pathogen throughout infection, with detectable cells in primary lesions for up to 28 days.^42^

Even before complete internalization, the capsule functions as the fungus’s first line of defense. In DCs, this component plays a central role by blocking cellular maturation, a process that is crucial for the activation of T cell-mediated adaptive immunity.^86^ Once internalized, the yeast resides within a *Cryptococcus*-containing vacuole (CnCV). Under physiological conditions, phagosomal maturation depends on coordinated fusion and fission events with endocytic organelles, regulated by small GTPases of the Rab family.^44^ *Cryptococcus*, however, actively manipulates the acquisition and retention of these proteins, preventing the formation of a fully degradative phagolysosome.^87^ Kinetic studies show that within the first 5 min after uptake, RAB5 (an early phagosome marker) is recruited. Yet, by 15 min, CnCVs prematurely lose RAB5 and RAB11 (an endosomal recycling marker), thereby interrupting canonical maturation progression. In contrast, at 120 min, anomalous retention of Rab9 is observed in CnCVs containing viable fungi.^44^ Because RAB9 is typically associated with trafficking between late endosomes and the trans-Golgi network, these findings suggest that the fungus reroutes membrane trafficking to generate a chimeric, protective compartment.^88^

Phagosomal acidification is a central effector mechanism because it activates hydrolytic proteases essential for intracellular killing. To counteract this process, *Cryptococcus* employs urease, which catalyzes urea hydrolysis into ammonia and carbon dioxide; upon protonation within the phagosomal lumen, ammonia acts as a buffer and elevates pH.^47^ In macrophages, *ure1*Δ*m*utants form CnCVs that are significantly more acidic than those containing the wild-type strain. Interestingly, although the fungus displays a higher in vitro growth rate at acidic pH, partial pH neutralization in vivo induces a strategic delay in intracellular replication, minimizing early damage to the host cell and enabling prolonged persistence within macrophages.^89^ This strategy supports systemic dissemination by preserving macrophage integrity, allowing these cells to function as transport vehicles across biological barriers, including the BBB.^46^ In addition, *Cryptococcus* disrupts intraphagosomal calcium homeostasis.^42,44^ Since calcium is an indispensable second messenger for vesicular fusion progression, its reduction in CnCVs harboring viable fungi constitutes an additional blockade to maturation. Thus, elevated pH, low proteolytic activity, and reduced calcium converge to convert the CnCV into a safe refuge for the yeast.

A particularly notable evasion mechanism described in the hypervirulent *C. gattii* R265 lineage is the formation of an “F-actin cage” around the phagosome in DCs.^45^ In general, actin polymerization is required only during engulfment and is rapidly depolymerized to permit phagosomal maturation.^44^ In contrast, strain R265 induces persistent retention of filamentous actin, surrounding the CnCV with a dense, branched structure detectable only by super-resolution microscopy. This cage acts as a physical barrier to lysosomal approach and fusion, blocking acidification and intracellular killing, resulting in high fungal burdens within DCs. Beyond protection, its persistence is linked to immune paralysis of these cells. The treatment with cytochalasin D (an actin inhibitor) disrupts the cage and rapidly restores phagosomal maturation, acidification, and the capacity to activate T cells. Collectively, this mechanism indicates that cytoskeletal manipulation is an active strategy to “hide” the compartment of antigen recognition and processing.^45^ Importantly, although this phenotype is well documented in the hypervirulent *C. gattii* R265 lineage, its distribution across other *C. gattii* lineages and related cryptococcal species remains uncertain, and the fungal determinants that initiate and maintain this actin-based phagosomal remodeling are still incompletely understood.

Over time, prolonged survival and replication within the CnCV compromise phagosomal membrane integrity through a process termed phagolysosomal membrane permeabilization (PMP). PMP enables leakage of luminal contents into the cytosol, promoting nutrient access and contributing to host programmed cell death. Two fungal virulence factors stand out: phospholipase B1 (PLB1) and physical capsule enlargement. PLB1 chemically destabilizes the lipid bilayer by hydrolyzing phospholipids,^48^ whereas volumetric capsule growth imposes substantial mechanical pressure within the confined phagosomal space. PMP is strongly associated with apoptosis induction because the leakage of luminal cathepsins activates caspase cascades, although extensive damage may also culminate in caspase-independent cell death pathways.^48^ Macrophages undergoing PMP lose the ability to maintain acidic pH and tend to undergo lytic exocytosis, releasing viable yeasts into the extracellular milieu; conversely, cells with preserved membranes favor nonlytic exocytosis (vomocytosis).

Uniquely, *Cryptococcus* not only avoids autophagic degradation but also subverts this pathway to facilitate trafficking and replication. Functional genomic screens indicated that infection activates the Autophagy Initiation Complex, involving the regulators LKB1 and AMPKα1.^90^ Proteins such as Atg5, Atg9a, and LC3 are recruited to the vicinity of CnCVs at early stages, and conversion of LC3-I to LC3-II is strongly induced by fungal presence. Consistently, RNAi-mediated silencing of autophagy components or inhibition of class III PI3-kinase drastically reduces the ability of *Cryptococcus* to replicate and escape from host cells. These data support that Atg protein-mediated trafficking is required to guide the CnCV toward a terminal compartment favorable for proliferation, converting a cellular surveillance system into a facilitator of virulence.^43^

Finally, escape from the intracellular niche can occur either via host cell lysis or through a nonlytic, highly coordinated process termed vomocytosis. This mechanism allows the yeast to exit macrophages without compromising host cell viability and is regulated by host and fungal factors, including urease and PLB1. By modulating phagosomal pH, urease promotes vomocytosis; absence of the enzyme significantly reduces the frequency of these “silent” escape events.^46^ Maintenance of a less acidic pH appears to signal conditions permissive for CnCV fusion with the plasma membrane. Thus, nonlytic exocytosis consolidates as a key dissemination strategy. The fungus can transit between cells and reach the bloodstream without triggering massive inflammation, and once in circulation, yeasts within migratory macrophages can cross the BBB, culminating in CNS invasion.^91,92^

In summary, *Cryptococcus*’s ability to remodel the phagosomal environment, transforming a compartment destined for degradation into a protected and nutritive niche, constitutes the central pillar of its intracellular pathogenesis (Figure 3). By orchestrating a sophisticated balance between the subversion of vesicular maturation, pH buffering, and cytoskeleton manipulation, the fungus not only ensures its survival and replication but also redefines the role of macrophages and DCs. These cells, originally sentinels, begin to act as ″Trojan horses″ that facilitate silent persistence and subsequent systemic dissemination. The transition between CnCV and strategic exit via vomocytosis exemplifies a refined coevolution, where the control of intracellular homeostasis dictates the pace of central nervous system (CNS) invasion. Understanding the fungal determinants that govern these intracellular plasticity remains, therefore, essential for the development of new therapeutic strategies aimed at interrupting the dissemination cycle of this pathogen.

**Figure 3:**
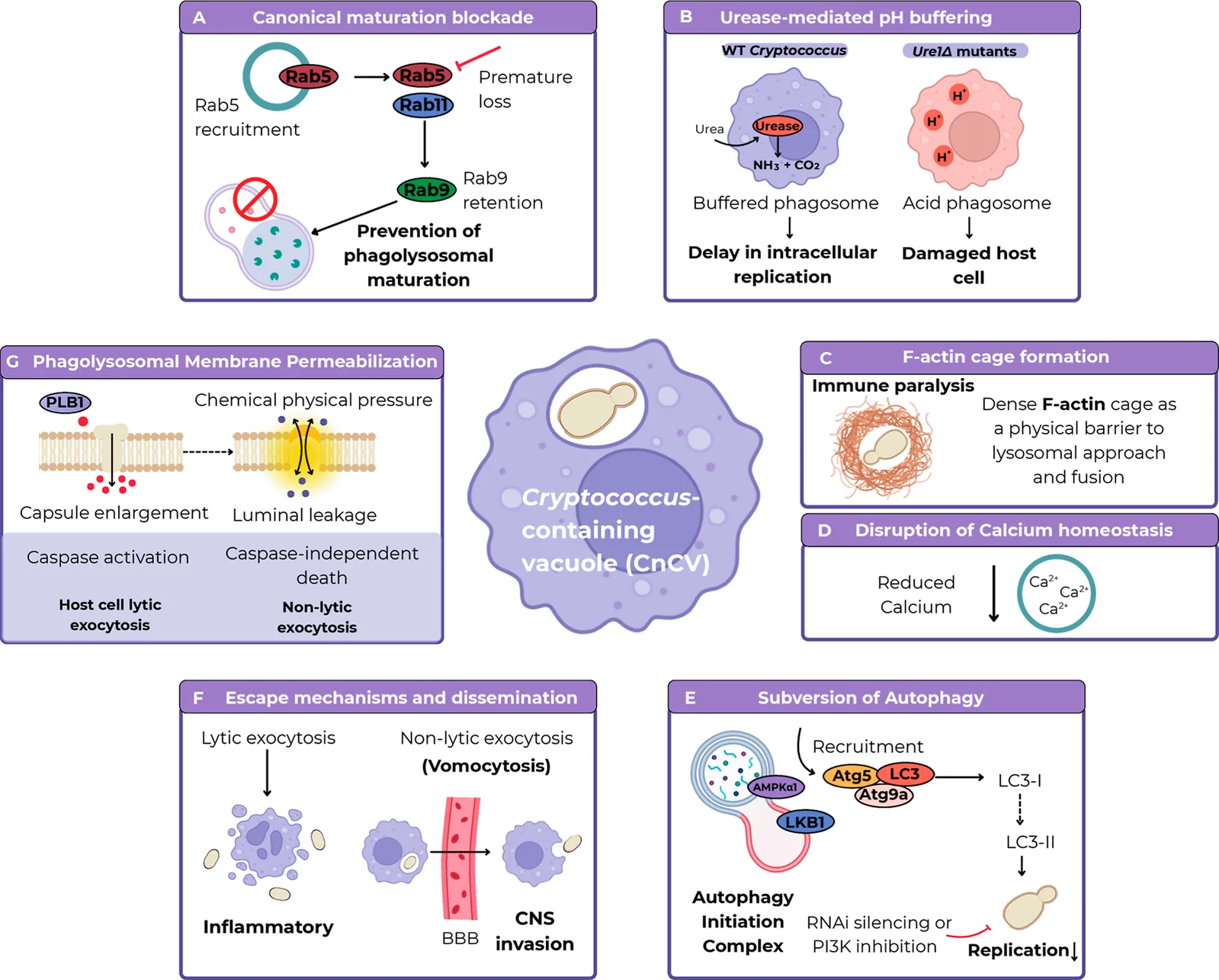
Overview of the mechanisms of intracellular niche manipulation and the phagosome. (A) Canonical blockade of phagosomal maturation: The fungus prevents the progression of the endocytic pathway by promoting the premature loss of early phagosome markers (such as Rab5 and Rab11) and inducing anomalous retention of Rab9, preventing phagolysosomal fusion; (B) urease-mediated pH buffering: Fungal urease activity converts urea to NH_3_ and CO_2_, buffering the phagosomal lumen. In wild-type (WT) strains, this results in a buffered phagosome and delayed intracellular replication, while *ure1*Δmutants face an acidic environment, leading to host cell damage; (C) formation of an F-actin ″cage″: The induction of a dense network of filamentous actin (F-actin) around the CnCV serves as a physical barrier, preventing lysosomal access and fusion, contributing to immune paralysis; (D) disruption of calcium homeostasis: The fungus induces a reduction in Ca^2+^ levels in the CnCV, interfering with calcium-dependent vesicular fusion processes; (E) subversion of autophagy: *Cryptococcus* manipulates the Autophagy Initiation Complex (involving AMPKα1 and LKB1), recruiting key proteins such as Atg5, Atg9a, and LC3, converting LC3-I to LC3-II to create a terminal favorable to its proliferation. RNAi silencing or PI3K inhibition can block this process; (F) escape and dissemination mechanisms: Yeast exit can occur through lytic (inflammatory) exocytosis or nonlytic exocytosis (vomocytosis). Vomocytosis facilitates systemic dissemination and crossing the BBB for invasion of the CNS; (G) phagolysosomal membrane permeabilization (PMP): factors such as capsule enlargement and the action of PLB1 exert physical and chemical pressure, respectively, on the phagosome membrane, causing leakage of luminal contents and culminating in PMP. Depending on the extent of damage and caspase activation, this leads to lytic or nonlytic cell death. Abbreviations: CnCV, *Cryptococcus*-containing vacuole; BBB, blood–brain barrier; CNS, central nervous system; PMP, phagolysosomal membrane permeabilization. Illustration created by the authors using Biorender (https://www.biorender.com/) and Canva (https://www.canva.com/). Graphical elements were used under valid publication licenses in accordance with the respective platform license agreements. Therefore, no additional permission from third-party copyright holders was required.

### 2.4 Nonlytic Exit and Systemic Dissemination

The identity of the inhaled fungal particle, spore or vegetative yeast, alters disease outcome and dissemination kinetics. Although both forms can cause fatal infection, spores display a superior capacity to colonize the CNS, resulting in brain fungal burdens approximately 18-fold higher than those observed in yeast-initiated infections at the time of death.^54^ This difference is even more evident when strains that are avirulent in the yeast form are analyzed. Spores derived from these same avirulent parents are fully capable of causing fatal meningoencephalitis in murine models.^54^ This phenomenon suggests that intrinsic properties of spores, such as the presence of surface ligands that facilitate rapid and efficient phagocytosis without the need for opsonization, allow the fungus to access immunological niches that are inaccessible to the encapsulated yeast.^54^

The ability of *Cryptococcus* to move between biological compartments without triggering a fulminant inflammatory response is central to its survival and systemic dissemination. In this context, nonlytic exocytosis (vomocytosis) and lateral transfer between phagocytes (dragotcytosis) function as high-precision mechanisms, enabling the fungus to exploit immune cells themselves as “vehicles” to reach sites such as the CNS.^92^

Vomocytosis is a singular cellular process in which *Cryptococcus*, even when contained within a mature phagosome, is expelled to the extracellular milieu while preserving the integrity and viability of both the fungus and the host phagocyte.^55^ This phenomenon contrasts with classical cell lysis, which results from uncontrolled fungal replication and membrane rupture, causing the release of damage-associated molecules and triggering inflammatory cascades.^53^ Thus, vomocytosis allows the pathogen to “reset” its anatomical position and explore new niches without alerting the adaptive immune system.^49^

Based on light and electron microscopy analyses, these events have been subdivided into three categories reflecting distinct states of fungus-host interaction.^49^ In type I, the macrophage expels the entire fungal burden at once in a coordinated event in which the phagosome migrates to the cell periphery and fuses with the plasma membrane. In type II, partial expulsion occurs, whereby the phagosome can undergo fission, releasing only a fraction of fungal cells while retaining the remainder, thereby sustaining continued intracellular replication. In type III, termed dragotcytosis, the yeast is transferred directly from an infected phagocyte to an adjacent cell, reducing prolonged exposure to the extracellular environment. The kinetics of these events are heterogeneous. Type I and II expulsions tend to occur after several hours of intracellular residence, while type III manifests earlier during infection.^93^ This early onset suggests a biological priority for tissue dissemination, as rapid movement between immune “sentinels” increases the likelihood of encountering more permissive environments.^55^

Among the most sophisticated regulatory mechanisms, governing vomocytosis is modulation of the host actin cytoskeleton. The *Cryptococcus*-containing phagosome is dynamic and can undergo repeated cycles of actin polymerization (“actin flashes”), characterized by transient cages of filamentous actin surrounding the compartment.^49^ These flashes are observed in murine and human macrophages and consist of dynamic filamentous actin cages enveloping the phagosome. Unlike pathogens such as *Listeria monocytogenes*, which exploit actin for propulsion and dissemination,^94^ in *Cryptococcus* these flashes appear to exert an inhibitory/containment function.^50^ Fluorescence-based assays and pharmacological approaches reveal an inverse relationship between flash frequency and vomocytosis occurrence; actin stabilization prolongs cage persistence and suppresses expulsion, whereas inhibition of actin polymerization or the Arp2/3 complex markedly increases nonlytic escape rates.^49^

This axis connects to the integrity of the phagosomal membrane, where approximately 95% of phagosomes containing the fungus exhibit some degree of integrity loss before vomocytosis, evidenced by the leakage of dextrans into the cytosol.^95^ The macrophage appears to respond with rapid actin polymerization mediated by Arp2/3 and WASP/N-WASP, attempting to “seal” the compartment and prevent phagosome-plasma membrane fusion. Thus, vomocytosis emerges as a critical balance point between immunological containment and fungal escape.

The decision to abandon the intracellular environment is also guided by stress sensing. Macrophages adopt a “bet-hedging” strategy in phagosomal acidification, generating a stochastic distribution of pH across phagocytes in which most reach a pH that is suboptimal for the fungus, whereas a fraction achieves particularly hostile levels.^55^ *Cryptococcus* monitors this microenvironment and tends to initiate exit programs when stress becomes unsustainable. Dragotcytosis, in particular, is associated with fungal damage induced by extreme acidity or severe oxidative stress, as indicated by lateral-transfer events occurring predominantly in phagolysosomes with low pH and high reactive oxygen species (ROS) burden. The degree of intracellular “hospitality,” in turn, depends strongly on the macrophage activation state. M1 macrophages constitute hostile niches, with higher inducible nitric oxide synthase expression and more stringent acidification; in these cells, fungal viability declines and vomocytosis/dragotcytosis frequency increases, reflecting the need to escape a fungicidal environment.^96^ In contrast, M2 macrophages are more permissive, exhibiting higher arginase-1 activity and phagosomes with a higher pH, closer to the optimum for *Cryptococcus* growth.^55^ In this context, the fungus replicates rapidly and the need for lateral escape is reduced. There is also evidence that infection may drive population-level skewing toward M2 polarization through secretion of proteins and manipulation of host signaling pathways, thereby shaping a maximally permissive niche and potentially dampening Th1 responses that are more effective in controlling infection.^97^

One major outcome of systemic dissemination is cryptococcal meningitis, which requires traversal of the BBB. Current evidence indicates that *Cryptococcus* does not rely on a single mechanism to reach the CNS, but rather exploits at least two major and partially complementary routes: direct traversal as a free fungal cell and transport within infected phagocytes through the “Trojan horse” pathway.^53^ A third possibility, the paracellular route, has also been proposed in the literature, although the strongest mechanistic support currently centers on transcellular traversal and phagocyte-assisted transport.^98^ Thus, BBB crossing should be interpreted as a flexible, context-dependent process rather than a fixed, uniform event.^51,54^ In the free-cell route, endothelial interaction is not passive, but depends on a coordinated molecular dialogue between fungal surface determinants and brain microvascular endothelial cells. A central axis in this process is the interaction between cryptococcal hyaluronic acid and the host receptor CD44. Hyaluronic acid expressed at the fungal surface promotes adhesion to brain microvascular endothelial cells, while CD44 on the endothelial membrane functions as a critical receptor for fungal attachment and uptake.^99^ Experimental evidence further indicates that CD44 deficiency is associated with reduced brain fungal burden, supporting the view that this receptor is functionally important for CNS invasion rather than merely permissive for adhesion. Downstream of this interaction, signaling pathways involving cytoskeletal remodeling are activated, favoring endocytosis-like uptake and transcellular passage across the BBB.^53^

This direct endothelial route is further strengthened by the secreted metalloprotease Mpr1, which has emerged as a key fungal determinant of BBB penetration. Mpr1 facilitates fungal association with brain endothelial cells and promotes transcytosis through mechanisms linked to host cytoskeletal remodeling. Importantly, experimental report has shown that expression of cryptococcal Mpr1 is sufficient to confer BBB-crossing capacity to heterologous yeast, underscoring that this protease is not merely correlated with neuroinvasion but is mechanistically active in the process.^100^ Additional evidence indicates that Mpr1 function involves Annexin A2 (AnxA2)-dependent trafficking events and promotes endothelial remodeling that supports transcellular fungal passage.^101^ Taken together, the hyaluronic acid/CD44 axis and the Mpr1–AnxA2 pathway indicate that free-cell transcytosis is an active invasion program requiring both fungal ligands and host-cell reprogramming.^99^

By contrast, the Trojan horse mechanism allows *Cryptococcus* to cross the BBB while sheltered within host phagocytes, particularly monocytes and macrophages. In this model, infected phagocytes traverse the endothelial barrier without major disruption of transendothelial electrical resistance, preserving barrier integrity while delivering viable fungal cells to the CNS.^91^ This route offers several potential advantages: protection from serum-mediated stress, partial concealment from circulating immune effectors, and coordinated transport through physiological migratory programs of host leukocytes. Direct imaging and in vitro BBB models support a significant contribution of Trojan horse transit to cryptococcal brain entry, reinforcing the idea that intracellular survival is not only a persistence strategy but also a dissemination strategy.^91^

Rather than treating these mechanisms as mutually exclusive, it is more accurate to consider that distinct biological contexts may favor one route over the other. Free-cell transcytosis is likely favored when fungal cells efficiently engage endothelial receptors, express the appropriate invasive determinants, and encounter a BBB environment permissive to receptor-mediated uptake and membrane trafficking. This route is especially consistent with strong endothelial adhesion, CD44-dependent signaling, and Mpr1-driven cytoskeletal remodeling.^101^ In contrast, Trojan horse dissemination may be favored when fungal cells are already established within permissive phagocytes, when systemic inflammation enhances leukocyte–endothelium interactions, or when intracellular carriage provides a selective advantage against extracellular immune pressures.^53^ In this sense, the choice of route may reflect not only fungal genotype and virulence-factor expression, but also host inflammatory status, phagocyte activation state, and the relative fitness costs of extracellular versus intracellular dissemination.

A critical comparative point is that the two mechanisms solve different biological problems. Free-cell transcytosis requires successful endothelial invasion and active manipulation of host trafficking pathways, but avoids the constraints of intracellular residence.^99^ Trojan horse transport, in turn, depends on prior survival within phagocytes and on host-cell migration across the BBB, but provides protection during bloodstream transit and may allow fungal cells to reach the brain in a more immunologically concealed state.^102^ Therefore, the most plausible model is not an “either/or” scenario, but a mixed framework in which *Cryptococcus* exploits both direct endothelial crossing and phagocyte-assisted transport according to infection stage and host microenvironment. Framing BBB traversal in this way strengthens the interpretation of neuroinvasion as an extension of immune evasion: the fungus reaches the brain not by a single stereotyped pathway, but by flexibly coupling virulence determinants, host-cell manipulation, and dissemination niche.

Extending the concept of vomocytosis to DCs and neutrophils further reinforces that *Cryptococcus* has co-opted fundamental, conserved processes of the innate immune system.^103^ Murine bone marrow, derived DCs undergo nonlytic exocytosis with rates and kinetics similar to those of macrophages, an observation of particular relevance because DCs migrate to draining lymph nodes for antigen presentation; by exploiting this pathway, the fungus may gain early access to the lymphatic system and systemic circulation. Inhibition of vomocytosis in DCs by bafilomycin A supports phagosomal acidification as a universal regulator of the process across distinct myeloid lineages.^103^ Collectively, these data indicate that *Cryptococcus* does not rely on a single cell type, but rather exploits shared circuits across multiple phagocytic populations.^103^ Accordingly, dissemination should be understood as a continuous cycle: initial capture by alveolar macrophages or DCs in the lung,^103^ intracellular survival with phagosomal buffering,^52^ intracellular survival with phagosomal buffering,^49^ followed by vomocytosis and/or dragotcytosis^55,93^ and ultimately systemic transport associated with the migration of infected phagocytes.^52^

In conclusion, the systemic dissemination of *Cryptococcus* transcends mere passive survival, configuring itself as a multimodal and highly plastic biological program (Figure 4). The strategic alternation between intracellular persistence and controlled escape via vomocytosis or dragocytosis allows the fungus to subvert immune surveillance, transforming phagocytes into vectors of systemic transport. This sophistication reaches its peak in the crossing of the BBB, where the pathogen does not depend on a single pathway, but exploits the complementarity between the Trojan horse mechanism and the active transcytosis of free cells, the latter mediated by precise molecular axes such as hyaluronic acid/CD44 and the Mpr1 protease. Whether through the accentuated neurotropy of its spores or the dynamic manipulation of the host’s actin cytoskeleton, *Cryptococcus* coordinates a silent invasion that culminates in the colonization of the CNS. Understanding this transition from a local pulmonary event to a continuous cycle of evasion and migration is crucial for identifying new therapeutic targets capable of halting the fatal progression of cryptococcal meningoencephalitis.

**Figure 4:**
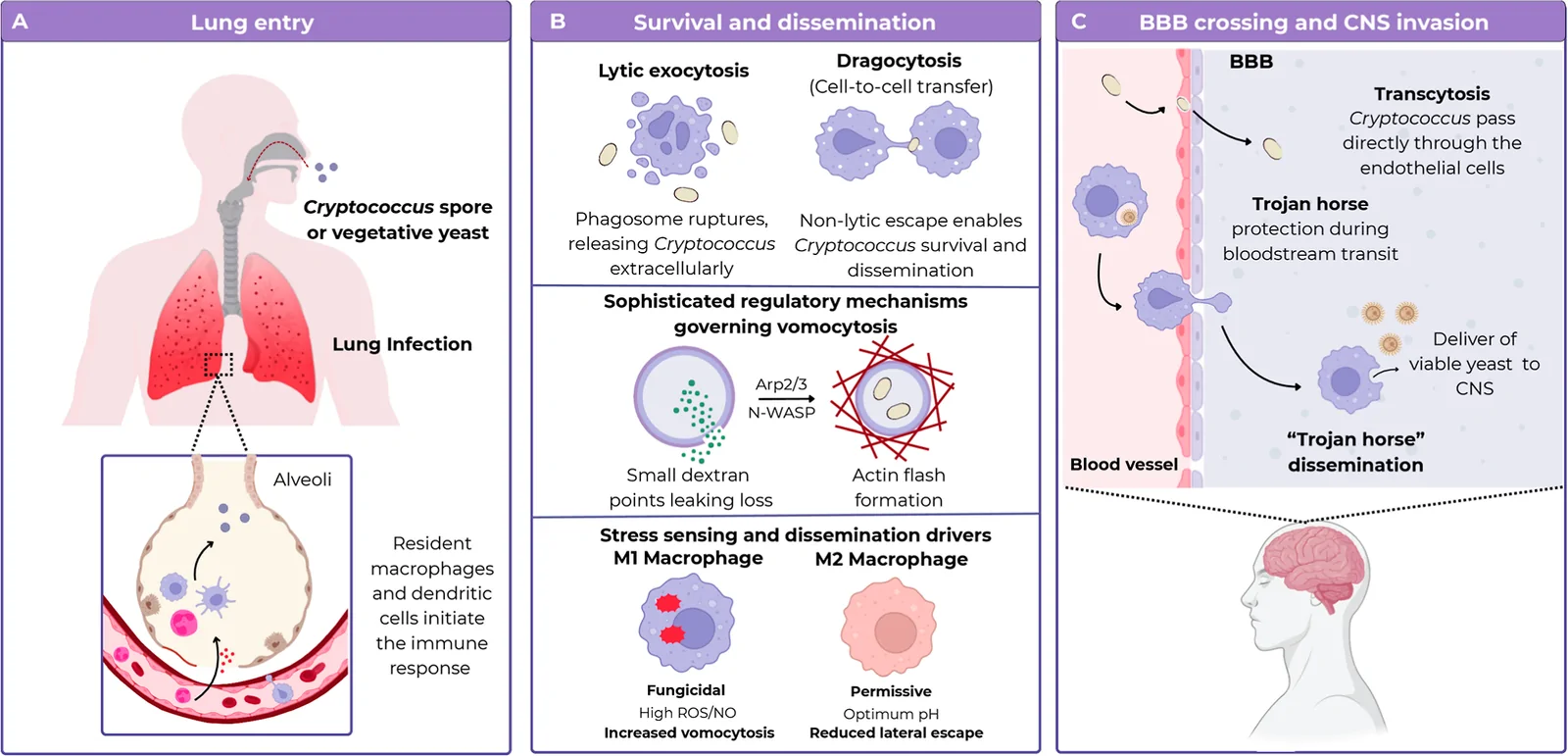
Multistaged mechanisms of *Cryptococcus* infection, intracellular survival, and neuroinvasion. (A) Lung entry: Inhaled spores or vegetative yeast cells reach the alveolar space, where they are internalized by resident alveolar macrophages and dendritic cells. (B) Phagosomal dynamics and dissemination: Contrast between lytic exocytosis (leading to host cell rupture) and vomocytosis (nonlytic exocytosis). Regulatory mechanisms of vomocytosis involve the formation of transient actin cages (″actin flashes″) mediated by Arp2/3 and WASP/N-WASP to contain the pathogen, and the sensing of phagosomal integrity loss via dextran leakage. The decision for fungal escape is driven by the host’s activation state: M1 macrophages (fungicidal, high ROS/NO) promote higher rates of vomocytosis/lateral transfer, whereas M2 macrophages (permissive, optimum pH) favor intracellular replication. (C) Blood–brain barrier (BBB) crossing: Systemic dissemination leads to CNS invasion through the ″Trojan horse″ mechanism, where infected phagocytes traverse the endothelial barrier, delivering viable yeast cells to the brain while remaining sheltered from systemic immune pressures. Illustration created by the authors using Biorender (https://www.biorender.com/) and Canva (https://www.canva.com/). Graphical elements were used under valid publication licenses in accordance with the respective platform license agreements. Therefore, no additional permission from third-party copyright holders was required.

### 2.5 Morphological Changes and Heterogeneity

The ability of *Cryptococcus* to survive within the hostile host environment is also supported by phenotypic flexibility expressed through morphological plasticity and population heterogeneity. Unlike many pathogenic fungi that rely on simple dimorphic transitions, the *Cryptococcus* species complex mobilizes a spectrum of cellular forms, including titan cells, senescent cells, dormant yeasts, and mucoid variants, to sustain chronic infections and to promote dissemination to the CNS.^104^ Thus, this plasticity is not merely a passive response to stress, but a coordinated adaptation strategy across distinct host niches, with direct consequences for evasion of immune recognition.^105^

“Titanization” stands out as one of the most striking phenotypic shifts.^76^ After inhalation and arrival in the pulmonary environment, yeasts of typical size (5–7 μm) can differentiate into giant cells (titan cells), with cell diameters exceeding 15 μm and often reaching up to 100 μm.^57^ This transition is induced by host-derived signals such as physiological CO_2_ levels, hypoxia, nutritional stress, serum exposure, and quorum-sensing molecules, integrated predominantly through the cAMP-PKA pathway.^85,104^ Biochemical profiling by high-performance liquid chromatography (HPLC) revealed that the polysaccharide composition of titan cells generated in vivo differs markedly from both cells grown in vitro and typical cells recovered from the same infectious microenvironment.^57^ Increased glucosamine was observed, suggesting enhanced deposition of chitin and chitosan in the inner cell wall layer, accompanied by a proportional reduction in glucose, consistent with a relative decrease in surface glucans.^57^ This remodeling has immunological implications because, at high levels, chitin can act as an immunemodulator, and its recognition by host chitotriosidase promotes Th2 polarization (IL-4 and IL-13), which is associated with a less effective antifungal response and may contribute to progression of pulmonary disease.^57,106^

A central component of titan cell-mediated evasion is masking of immunogenic patterns, particularly β-1,3-glucans. The host recognizes these ligands through Dectin-1 on macrophages and DCs, initiating a robust pro-inflammatory response.^57^ However, titan cell architecture, characterized by a markedly thick cell wall and a highly dense, cross-linked capsule, creates a physical barrier that limits Dectin-1 access to its ligands.^107^ This capsular density also reduces permeability to larger molecules such as complement proteins and antibodies, thereby diminishing lytic attacks and efficient opsonization.^107^ In addition, mannose fibrils on the outer surface of the wall, observed by electron microscopy, suggest an extra protective layer that distinguishes these cells from smaller and younger morphotypes.^57^

A particularly relevant aspect is that titan cells may provide “cross-protection” to surrounding populations of typically sized yeasts.^85^ Due to their size, they are largely refractory to phagocytosis by alveolar macrophages.^107^ Yet, their impact appears to extend beyond intrinsic resistance, as their presence can reduce phagocytosis of smaller cells, potentially through robust secretion of soluble capsular polysaccharides (inducing local immune paralysis) and/or interference with pattern-recognition signaling.^104^

*Cryptococcus* pathogenesis also involves prolonged latency, consistent with refined mechanisms governing entry into and exit from metabolically dormant states.^58^ Heterogeneity within the host is not only morphological but also metabolic, with subpopulations coexisting within the same tissue and exhibiting distinct replication rates and stress responses.^108^ In flow-cytometry studies, *C. neoformans* subpopulations were discriminated by enzymatic activity and glutathione levels using the fluorescent probe CMFDA (5-chloromethylfluorescein diacetate).^57^ Whereas a large fraction of cells early in infection is CMFDA-high (metabolically active), a CMFDA-low fraction gradually emerges, associated with a “viable but non-culturable” or dormant state.^58^ Although these cells show limited growth on conventional laboratory media, they can be “rescued” and induced to resume proliferation upon exposure to fetal bovine serum, interpreted as a signal of a nutritionally favorable environment or of potential systemic dissemination.^108^

At the molecular level, dormancy involves specific adaptations such as upregulation of PCK1 (phosphoenolpyruvate carboxykinase), indicating reliance on gluconeogenesis to maintain viability under nutrient deprivation and/or intracellular stress,^58^ while downregulation of COX1 (cytochrome *c* oxidase subunit 1) suggests “mitochondrial quiescence,” reducing endogenous ROS and conserving energy for prolonged survival.^108^ This profile is particularly relevant to the Trojan horse strategy, as dormant yeasts can persist within macrophages and DCs and use them as vehicles to cross the BBB without fully activating immune surveillance.^58^ Under immunosuppressive conditions, reactivation of these cells promotes intense fungal proliferation and fatal meningitis.^104^

Heterogeneity is also shaped by replicative age. Because reproduction occurs by asymmetric budding, the mother cell accumulates bud scars and cellular damage across generations, leading to replicative senescence.^57^ Senescent *C. neoformans* cells (18–30 generations) exhibit increased cell body size and thicker capsules, resembling titan cells, although in this case the origin is aging rather than a host-induced differentiation program.^57^ An important finding is the link between senescence and genomic instability, which increases the rate of phenotypic switching.^57^ In particular, senescent RC-2 cells display up to an 11-fold increase in switching to the mucoid phenotype, characterized by glossy colonies enriched in capsular polysaccharides and associated with hypervirulence and enhanced resistance to innate immunity.^56^

Ultimately, this plasticity and heterogeneity depend on a dynamic structural scaffold within the cell wall. High-resolution solid-state NMR structural biology data indicate that the *Cryptococcus* wall is a complex matrix in which different glucan forms and chitin cooperate to provide both protection and flexibility, with α-1,3-glucan acting as a key component.^109^ Beyond its structural role, this polymer is indispensable for virulence by serving as an anchor for the capsule on the outer surface. Cells unable to produce α-1,3-glucan can synthesize capsular components but fail to retain them, resulting in effectively acapsular cells that are rapidly cleared by the immune system.^57^ Under carbon starvation, a recurrent stress during infection, concomitant remodeling of capsule and wall is observed, with increased porosity and permeability mediated by chitin synthases and β-glucan-specific glycosidases, suggesting surface reorganization to optimize nutrient acquisition without complete loss of physical protection.^110^

Finally, plasticity and heterogeneity converge toward continuous microevolution during chronic infections.^105^ Serial isolates from patients with recurrent meningitis reveal relevant genomic restructuring, including aneuploidies, gene duplications, and point mutations in regulators of virulence and resistance.^111^ Among the observed patterns are (i) amplification of resistance genes, such as duplication of erg11 under fluconazole pressure;^112^ (ii) mutations in transcription factors, such as alterations in GAT20, associated with phagocytosis regulation, which progressively refine macrophage evasion;^113^ and (iii) tissue-specific adaptations, in which pulmonary cells tend to favor titanization to resist early phagocytosis, whereas CNS-resident cells may adopt smaller morphologies and metabolic programs compatible with increased inositol utilization, abundant in the brain and associated with enhanced capsule production and cellular invasion.^105^ The coexistence of genetically and phenotypically distinct sublineages within the same patient suggests diversifying selection and niche partitioning, ensuring that even when one subpopulation is controlled by immunity or antifungal therapy, others persist in tissue reservoirs and sustain clinical relapses and the chronic nature of cryptococcosis.^105^

Ultimately, the virulence of *Cryptococcus* does not reside in a static cellular state, but in its extraordinary ability to act as a ″biological chameleon.″ The coexistence of distinct morphotypes, from the imposing titan cells that paralyze the pulmonary immune response to the dormant yeasts that silently persist in metabolic niches, reflects a sophisticated strategy of niche partitioning and bet-hedging. This heterogeneity, fueled by a dynamic cell wall architecture (rich in α-1,3-glucan and chitin) and by genomic microevolution processes, ensures that the pathogen remains a mobile target for the immune system and antifungal therapies. Therefore, the chronicity of infection and the recurrence of meningitis are not mere byproducts of host failure, but the direct result of programmed plasticity that allows the fungus to exploit the vulnerability of each biological compartment (Figure 5). Deciphering the molecular triggers of this versatility is, therefore, the next critical step in transforming the clinical management of a pathology marked by latency and fatal relapses.

**Figure 5:**
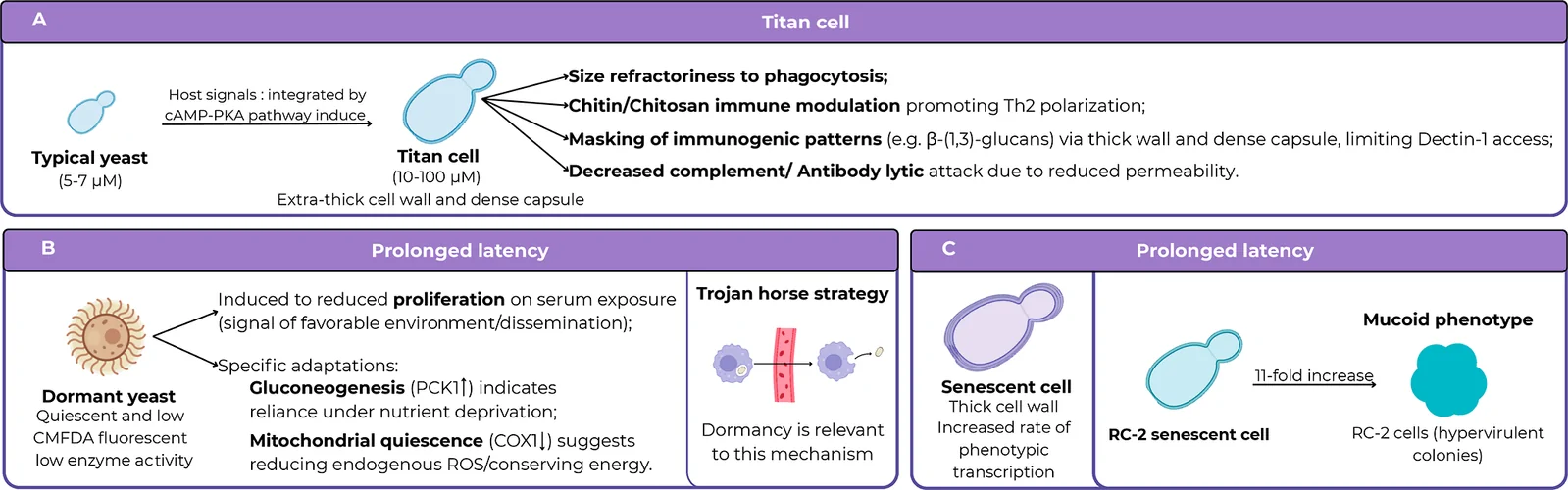
Morphological plasticity and population heterogeneity as drivers of *Cryptococcus* immune evasion and persistence. (A) Titanization: Host-derived signals integrated via the cAMP-PKA pathway induce typical yeast cells (5–7 μm) to differentiate into giant Titan cells (>15 μm). Key features include an extra-thick cell wall and a highly dense capsule, which promote size-dependent refractoriness to phagocytosis; Th2 immune polarization through increased chitin/chitosan deposition; masking of immunogenic β-(1,3)-glucans from Dectin-1 recognition; and reduced permeability to complement proteins and antibodies. (B) Prolonged latency and metabolic dormancy: Dormant yeasts, characterized by low CMFDA fluorescence and enzymatic activity, undergo metabolic reprogramming to survive nutrient deprivation. Upregulation of PCK1 (gluconeogenesis) and downregulation of COX1 (mitochondrial quiescence) allow prolonged survival within host phagocytes, facilitating the ″Trojan horse″ dissemination strategy. (C) Senescence and phenotypic switching: Replicative aging leads to senescent cells with thickened cell walls and increased rates of phenotypic transcription. In the RC-2 strain, senescent cells exhibit an 11-fold increase in switching to the hypervirulent mucoid phenotype, characterized by colonies enriched in capsular polysaccharides and enhanced resistance to innate immunity. Illustration created by the authors using Biorender (https://www.biorender.com/) and Canva (https://www.canva.com/). Graphical elements were used under valid publication licenses in accordance with the respective platform license agreements. Therefore, no additional permission from third-party copyright holders was required.

### 2.6 Metabolic and Antioxidant Resistance

Upon inhalation by the host, *Cryptococcus* is immediately confronted by alveolar macrophages, which constitute the first line of immune defense in the lung.^59^ These cells activate a range of effector mechanisms, including the production of ROS and reactive nitrogen species (RNS), to eliminate the invader.^60^ In this context, the fungus’s antioxidant resistance becomes an indispensable condition for intracellular survival and establishment of primary infection.^114^

Among the most critical determinants of virulence is melanin, a pigment synthesized from exogenous phenolic compounds through laccase activity,^115^ and widely recognized as essential for *Cryptococcus* pathogenicity.^10^ CNLAC1 (also referred to as LAC1) encodes the principal laccase responsible for melanization, although the homologue LAC2 also contributes to this process.^59,116,117^ Melanin acts as a multifunctional shield; its polymeric structure confers physical and chemical resistance, preserving the integrity of the cell wall against enzymatic degradation and attack by antimicrobial peptides.^118^ In addition, it acts as a potent antioxidant capable of neutralizing free radicals generated by macrophages and neutrophils,^119^ which is reflected in the markedly increased resistance to macrophage-mediated killing observed in melanized strains compared with nonmelanized strains.^59^

The relevance of laccase, however, is not limited to the antioxidant role associated with melanin. The enzyme also exhibits ferroxidase activity, converting Fe­(II) to Fe­(III), which is particularly important because ferrous iron can catalyze the Fenton reaction and generate highly toxic hydroxyl radicals from peroxides.^120^ By favoring maintenance of iron in a less reactive oxidized form, laccase indirectly limits oxidative damage during host interaction. This dual role places laccase at the intersection of virulence factor production, redox homeostasis, and tissue dissemination. Interestingly, although laccase is essential for extrapulmonary dissemination, it appears dispensable for early pulmonary persistence, suggesting a more critical role during tissue invasion and circulatory transit.^114^

Beyond serving as a classical virulence factor, melanization should be interpreted as a direct countermeasure against the oxidative burst mounted by host phagocytes. During early pulmonary infection and intracellular residence, *Cryptococcus* is exposed not only to ROS generated by macrophages, but also to oxidative injury associated with recruited neutrophils. In this context, melanin functions as a redox-buffering shield that absorbs and neutralizes toxic intermediates before they damage essential fungal structures. This protection is especially relevant at the cell surface, where melanization reinforces wall resilience while simultaneously reducing the effectiveness of oxidative and enzymatic antimicrobial mechanisms. Thus, melanization is not merely a structural phenotype, but a dynamic antioxidant interface that preserves viability under immune pressure. The central enzyme in this axis, laccase, also contributes to oxidative resistance through mechanisms that extend beyond pigment synthesis itself. By promoting the oxidation of Fe­(II) to Fe­(III), laccase may limit iron-driven hydroxyl radical formation and thereby reduce secondary oxidative damage generated in inflamed host niches. From an immunological standpoint, the melanin–laccase system therefore acts as a fungal defense module that diminishes susceptibility to phagocyte-mediated killing, preserves survival within hostile inflammatory microenvironments, and supports persistence during the transition from pulmonary colonization to extrapulmonary spread.

Beyond melanin, *Cryptococcus* possesses a sophisticated enzymatic network for detoxification of organic and inorganic peroxides, supported by glutathione peroxidases (GPX) and thiol peroxidases (TSA), which act in an integrated manner to maintain cytosolic and mitochondrial redox balance.^60^ The *C. neoformans* genome contains two glutathione peroxidase homologues, GPX1 and GPX2, which are differentially regulated in response to stress.^86^ Both catalyze peroxide reduction using reduced glutathione as the electron donor, producing glutathione disulfide as a byproduct.^121^ Expression of these genes is induced by organic hydroperoxides, whereas only GPX2 is induced by hydrogen peroxide.^122^ Mutants deficient in both enzymes display increased sensitivity to organic peroxides and reduced intramacrophage survival, although overall virulence in murine models remains comparable to the wild-type strain, possibly due to compensation by other antioxidant systems.^60^

Within this axis, the thiol peroxidase TSA1, a 2-cys peroxiredoxin dependent on the thioredoxin system, assumes an even more central role in pathogenesis. Unlike GPX, TSA1 is required for full virulence and for resistance to nitric oxide and hydrogen peroxide.^123^ Its absence triggers compensatory responses, with induction of other stress-related enzymes, including laccase, as an attempt to mitigate cellular damage.^124^ This metabolic “backup” highlights the robustness and functional redundancy of the pathogen’s defenses against host innate immunity.

In parallel, *Cryptococcus* virulence is energetically demanding, because capsular polysaccharide synthesis and pigment production require a continuous supply of ATP and metabolic precursors.^125^ Thus, the ability to monitor and adjust energetic status under hypoxia and nutrient limitation constitutes a fundamental strategy of metabolic resistance.^61^ In this setting, kinases such as IRK2 and IRK5 have been identified as central regulators of metabolism and virulence in *C. neoformans*.^61^ These enzymes are critical for survival during pulmonary and cerebral infections. Quantitative proteomics and untargeted metabolomics showed that deletion of IRK2 or IRK5 promotes profound cellular reorganization, notably including relevant changes in intracellular ATP levels, consistent with direct coordination of mitochondrial function.^59,61^ In particular, IRK5 is directly associated with melanin production; *irk5*Δ*m*utants exhibit a drastic reduction in pigment synthesis, compromising the ability to evade oxidative responses in the host.^126^ Accordingly, integration between regulatory kinases and mitochondrial function optimizes energy production to sustain growth and expression of virulence factors under stress, ensuring sufficient resources to resist phagocytosis and promote dissemination to the CNS.^61^

Metabolic resistance is also reflected in flexible utilization of carbon sources according to the colonized niche. SAGE studies using cells isolated from mouse lungs revealed upregulation of genes associated with the glyoxylate cycle, gluconeogenesis, and the utilization of alternative substrates such as acetate.^125^ A classic example is ACS1, which encodes acetyl-CoA synthetase, catalyzing conversion of acetate to acetyl-CoA, a central precursor for the TCA cycle as well as for fatty acid and melanin biosynthesis.^125^ Deletion of ACS1 reduces virulence, reinforcing that metabolic plasticity is not merely a survival requirement but an intrinsic component of the virulence arsenal.^127^ Moreover, the ability to use inositol, abundant in the mammalian brain, as both a sole carbon source and a signal for capsule formation supports the metabolic specialization underlying the characteristic neurotropism of the fungus.^128^

Finally, protein quality-control mechanisms also contribute to adaptation under stress and chemical pressure. The ATP-dependent unfoldase/translocase CLPX is part of a proteolytic complex essential for cellular proteostasis.^62^ Using proteomic approaches, CLPX was identified as a molecular signature of fluconazole resistance in laboratory-evolved strains and clinical isolates.^62^ Evidence indicates that CLPX maintains heme availability and the stability of key enzymes in the ergosterol pathway, including Erg11, Erg10, and Erg7. Disruption of CLPX, by gene deletion or pharmacological inhibition, reverses the resistance phenotype and restores fluconazole susceptibility, both in vitro and in vivo.^129^ This mechanism illustrates how fine regulation of critical metabolic pathways and maintenance of proteostasis promote pathogen persistence under intense biological and therapeutic pressure.^59^ Consistently, CLPX has also been implicated in resistance to thermal and oxidative stress, suggesting participation in a multifaceted survival response within the host.^62^

Although macrophage-centered models dominate the current literature on cryptococcal intracellular survival, interactions with neutrophils also deserve greater attention in the context of oxidative immune escape. Neutrophils contribute to host defense through rapid production of reactive oxygen intermediates, degranulation, and, in some settings, extrusion of neutrophil extracellular traps (NETs). For *Cryptococcus*, these mechanisms likely represent an additional layer of selective pressure acting on melanization, antioxidant enzyme systems, and surface architecture. The capacity of melanin to quench reactive species generated by both macrophages and neutrophils supports the view that resistance to oxidative killing is not restricted to one phagocyte type, but is relevant across distinct innate effector compartments.

At the same time, the specific relationship between cryptococcal surface properties and NET-mediated containment remains underdeveloped in the literature and should be explicitly recognized as a knowledge gap. Capsular size, polysaccharide release, melanization, and cell-size heterogeneity may all influence whether fungal cells are efficiently trapped, damaged, or instead remain relatively protected from neutrophil-mediated attack. Thus, future studies should address how cryptococcal morphotypes and virulence factors modulate neutrophil recruitment, NET formation, and fungal survival within NET-rich inflammatory environments. Incorporating this perspective strengthens the review by extending oxidative immune evasion beyond macrophages and highlighting an important frontier for mechanistic investigation. Taken together, these observations indicate that metabolic adaptation and antioxidant resistance in *Cryptococcus* are best understood as integrated defenses against multiple innate effector systems, rather than as responses restricted to macrophage-derived stress alone.

In conclusion, the metabolic and antioxidant resistance of *Cryptococcus* should not be seen as a series of isolated events, but as an integrated defense module, that confers exceptional evolutionary robustness to the pathogen (Figure 6). The synergy between the physicochemical shield of melanin, the enzymatic network of peroxidases, and the metabolic flexibility to exploit alternative carbon sources (such as inositol and acetate) allows the fungus to neutralize oxidative stress while sustaining the high-energy cost of virulence. This physiological resilience, coordinated by regulatory kinases and proteostasis mechanisms via CLPX, ensures survival not only in the confined environment of macrophage phagolysosomes, but also in the face of neutrophil aggressiveness and circulatory transit. As research progresses to fill the gaps in our understanding of the interaction with NETs, it becomes evident that disrupting this redox–metabolic axis represents one of the most promising and challenging avenues for developing therapies aimed at disarming the persistence and systemic invasion capabilities of this fungus.

**Figure 6:**
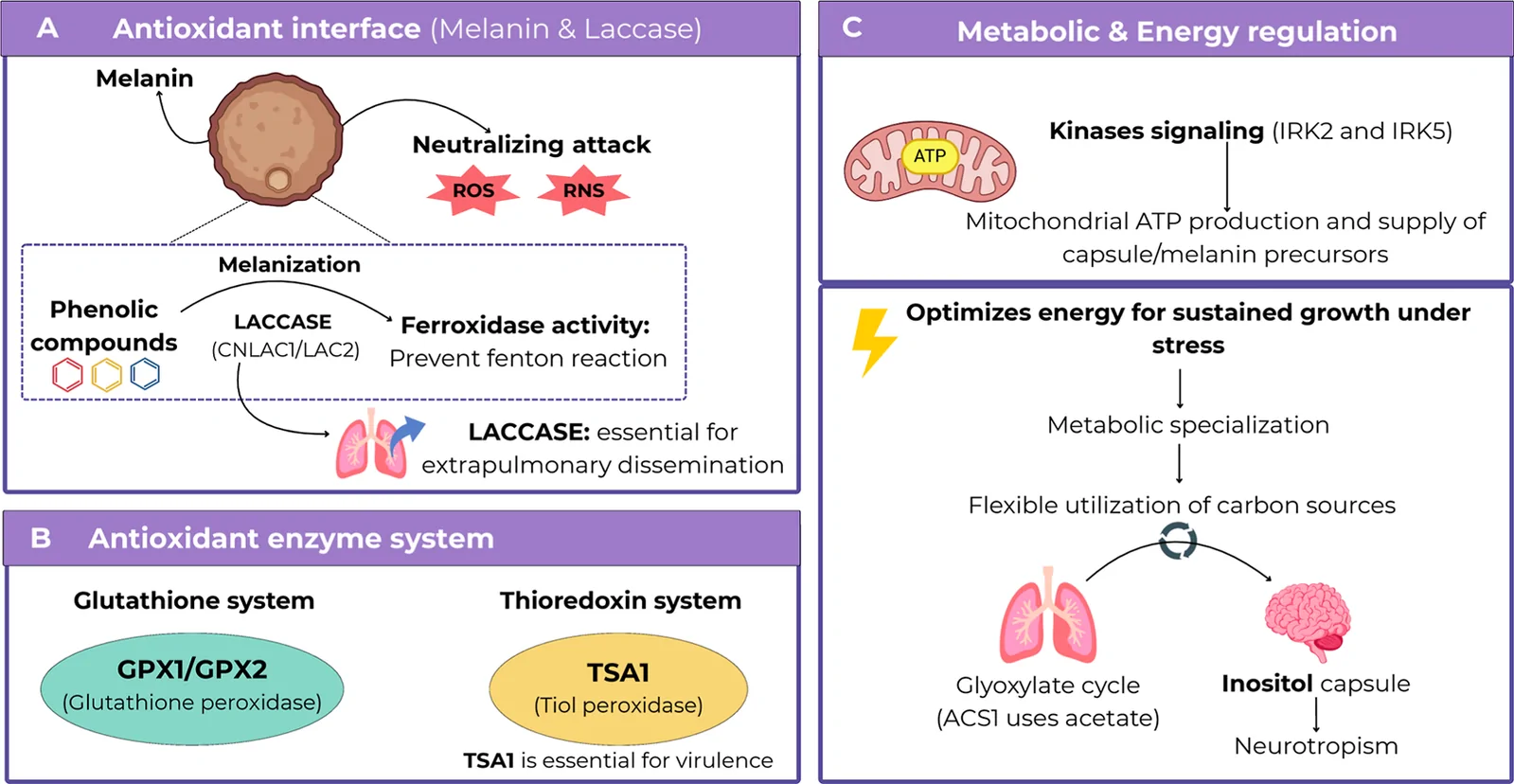
Overview of metabolic and antioxidant resistance mechanisms. (A) Melanization acts as a multifunctional shield, conferring physical resistance and neutralizing reactive oxygen species (ROS) and reactive nitrogen species (RNS) generated by the host. The enzyme laccase (CNLAC1/LAC2) catalyzes melanin synthesis and exhibits ferroxidase activity, preventing the Fenton reaction and limiting secondary oxidative damage. Laccase is essential for extrapulmonary dissemination; (B) *C. neoformans* possesses an enzymatic network for peroxide detoxification, supported by glutathione (GPX1/GPX2) and thioredoxin (TSA1) systems. The thiol peroxidase TSA1 plays a central role in pathogenesis, being necessary for complete virulence and resistance to oxidative and nitrosative stress; (C) IRK2 and IRK5 kinases coordinate mitochondrial function, ATP production, and the supply of precursors for the synthesis of virulence factors under stress. The flexibility in the use of carbon sources, such as the use of acetate by the glyoxylate cycle and inositol for the formation of the polysaccharide capsule, sustains the persistence and neurotropism characteristic of the fungus. Illustration created by the authors using Biorender (https://www.biorender.com/) and Canva (https://www.canva.com/). Graphical elements were used under valid publication licenses in accordance with the respective platform license agreements. Therefore, no additional permission from third-party copyright holders was required.

### 2.7 Interference with the Adaptive Immune Response

Orchestration of the adaptive immune response is a key determinant of clinical outcomes during infection. While innate immunity provides the first line of defense, durable containment and pathogen clearance depend on an efficient transition to cell-mediated immunity, sustained by lymphocyte activation and polarization toward a protective cytokine profile.^130^ However, *Cryptococcus* has evolved a repertoire of strategies capable of arresting, diverting, or subverting this response, thereby promoting survival and chronic persistence.^131^

This interference operates at multiple levels, beginning with disruption of antigen recognition, capture, and processing by DCs, progressing through manipulation of intracellular signaling in macrophages, and culminating in skewing of the balance among Th1, Th2, and Th17 responses.^132^ Classical virulence factors, such as the capsule and melanization, together with immunomodulatory enzymes, including phospholipase B1 (PLB1)^63^ and the DEAD-box RNA helicase VAD1,^64^ form a coordinated network that compromises lymphocyte clonal expansion and the effectiveness of immunological memory.

Initiation of a robust adaptive response requires that DCs capture fungal antigens, undergo maturation, and migrate to draining lymph nodes, where they present peptides via MHC to naïve T lymphocytes.^133^ *Cryptococcus* interferes early in this axis and, as noted above, xylose is a ubiquitous component of the pathogen’s surface glycans; recent studies indicate that xylosylation is a critical determinant of this interference. Mutant lineages deficient in xylose precursor transporters (*uxt1*Δ*a*nd *uxt2*Δ) show that, in the absence of proper xylosylation, the fungus loses the ability to suppress DC activation.^63^ Unlike the wild-type strain, which induces a muted response, these mutants stimulate DCs to produce significantly higher levels of pro-inflammatory cytokines, suggesting that xylose modulates PRR recognition and blocks signals required for protective polarization. Persistence of *uxt1*Δ*a*nd *uxt2*Δ*i*n the lungs of immunocompetent mice for more than 189 days, despite the lack of lethality, suggests containment by a T- and B-cell-rich response. By contrast, the early xylose-mediated interference exerted by wild-type strains appears to be a key factor preventing such containment.^63^

Suppression of cellular immunity has also been mapped by transcriptomic analyses that simultaneously assessed pathogen and host gene expression during interaction. Highly virulent *C. gattii* strains display a superior capacity to suppress core cell-mediated immunity genes in RAW264.7.1 macrophages.^69^ In these macrophages, downregulation was observed for genes associated with the transporter associated with antigen processing (TAP) complex and β2-microglobulin binding. Because TAP is essential for peptide loading onto MHC class I molecules, a key step for CD8+ T-cell recognition and cellular cytotoxicity, its inhibition contributes to a functional state of “immune blindness,” in which the adaptive response fails to identify infected cells.^134^ Moreover, highly virulent *C. gattii* strains inhibit M1 macrophage differentiation and favor transition to an M2 state permissive to fungal proliferation. This rerouting is supported by low expression of markers such as NOS2, TNF, and IL-6, together with upregulation of genes such as ARG1, IL-10, and CD163.1.^69^

An additional pillar of cryptococcal evasion is the manipulation of the Th1 versus Th2 axis. A Th1 response (IFN-γ and IL-12) is associated with fungal clearance, whereas a Th2 response is more often linked to disease progression and poorer prognosis.^64,131^ In this context, VAD1 emerges as a central global regulator of *C. neoformans* virulence, with profound impact on systemic and local adaptive immunity.^64^ In patients with cryptococcal meningitis, higher levels of VAD1 mRNA in cerebrospinal fluid correlate with lower IFN-γ and higher IL-10. During the acute phase, when fungal burden is highest, VAD1 reaches peak expression and promotes an IL-10-enriched profile, reducing macrophage effector activation and weakening cellular immunity in the CNS. With clinical stabilization, VAD1 decline parallels increases in IFN-γ/IL-10 and IL-12/IL-10 ratios. This effect appears to derive from control of fungal mRNA stability and, possibly, indirect interference with host signaling via the CCR4-NOT complex, which mediates transcriptional and post-transcriptional regulation.^135^

Chemical interference with adaptive immunity also occurs through secretion of bioactive lipids. *Cryptococcus* uses PLB1 to degrade phospholipids from host cell membranes and pulmonary surfactant, releasing arachidonic acid.^63^ This fatty acid can be metabolized by the fungus to inhibit lymphocyte proliferation and reduce chemokine production required for T-cell recruitment.^136^ In murine models, *plb1*Δmutants fail to induce pulmonary eosinophilia and permit a more protective cellular response capable of controlling fungal burden.^63^

Beyond cytokine skewing and lipid mediator modulation, *Cryptococcus* can directly limit clonal expansion of immune cells by interfering with proliferation and survival via NF-κB.^66^ Evidence indicates that *C. neoformans* infection reduces macrophage proliferation independently of the capsule, associated with cell-cycle disruption at the S and G2/M phases.^137^ This control of cell fate occurs through divergent modulation of NF-κB pathways. Proliferation arrest requires the canonical pathway, whereas induction of macrophage apoptosis is strictly dependent on the noncanonical pathway and on the presence of the polysaccharide capsule.^66^ The canonical pathway contributes to negative regulation of cyclin D1 and SKP2, culminating in growth arrest, whereas the noncanonical pathway is essential for pathogen-induced programmed cell death, enabling removal of immune cells without exacerbated inflammation.^65^ By uncoupling these routes, *Cryptococcus* simultaneously prevents amplification of adaptive defense and “silently” eliminates cells attempting to contain it.

Long-term containment and prevention of reactivation depend on memory T cells, yet the pathogen also interferes with the kinetics and functionality of this compartment, particularly tissue-resident memory T cells.^66^ During infection, CD4+ memory T cells increase rapidly as early as day 3, peaking on day 7 postinfection, with IFN-γ production relevant for initial containment.^68^ This accumulation depends on signaling through C-type lectin receptors via the adaptor protein CARD9.^68^ CARD9 deficiency results in a drastic reduction of IFN-γ-producing T cells and high host susceptibility. The fungus appears to engage receptors such as Dectin-2 through components including glucosylceramide and chitin. However, integration of signals via CARD9 determines whether tissue memory is efficiently activated or suppressed. In addition, memory-phenotype T cells can respond in a near-innate manner, even before classical consolidation of immunological memory.^138^ *Cryptococcus* exploits this temporal window, in which antigen-nonspecific tissue-resident T cells attempt to provide early protection. By interfering with maturation of these cells, the pathogen impairs establishment of a stable immunological barrier, favoring latency and reactivation.^68^

In the lungs, spatial organization of the adaptive response is centered on formation of inducible bronchus-associated lymphoid tissue (iBALT).^139^ Although cryptococcal infection induces iBALT, the pathogen manipulates its structural quality through capsular xylosylation.^66^ In infections with wild-type strains, iBALT forms rapidly but with small, compact B-cell follicles surrounded by disorganized T-cell zones. This architecture fails to restrict fungal burden, allowing dissemination through the parenchyma and disruption of tissue organization. In contrast, mutants with reduced xylose induce iBALT later, but in a significantly more organized and functional manner. In these cases, B-cell follicles are larger, T-cell zones are well demarcated, and sustained production of CXCL13, a chemokine essential for maintenance of lymphoid structure, is observed.^140^ Well-organized iBALT can physically confine mutant fungi for months, preventing systemic dissemination and suggesting that specific glycans are used to sabotage the “engineering” of local lymphoid tissue.^66^

Finally, brain invasion represents one of the greatest challenges to adaptive immunity, because the CNS is immunologically privileged and tightly regulates T-cell activation to prevent tissue damage.^141^ Massive deposition of GXM in cerebral vessels and in the subarachnoid space compromises leukocyte migration and endothelial function.^142^ Polysaccharide accumulation is associated with vascular occlusion, capillary thrombosis, and hemorrhages, facilitating hemorrhagic fungal dissemination.^67^ In patients with HIV and meningitis, minimal inflammation is observed due to CD4+ T-cell depletion; nevertheless, even in immunocompetent individuals, the fungus can evade an effective granulomatous response through profuse GXM secretion, which inhibits microglial phagocytosis and neutrophil chemotaxis.^143^

In summary, *Cryptococcus*’s interference with adaptive immunity represents one of the most refined facets of its survival strategy, operating as a multilevel sabotage of the host’s ″command center″ (Figure 7). By orchestrating a state of ″immunological blindness″, mediated by the inhibition of antigen processing via the TAP complex and the systematic shift to the Th2 profile, the pathogen prevents the consolidation of an effective cytotoxic and fungicidal response. The discovery that components such as capsular xylosylation and the VAD1 regulator can dictate everything from the structural disorganization of iBALT to the paralysis of resident memory cells reveals a pathogen that not only evades but also actively reconfigures the local and systemic immune architecture. Therefore, the transition to chronicity and the eventual invasion of the central nervous system are not mere surveillance failures, but the outcome of an active immunomodulation program. Understanding these mechanisms of immunological memory disruption is imperative for the design of vaccines and adjuvants that can restore the host’s ability to contain the advance of this persistent invader.

**Figure 7:**
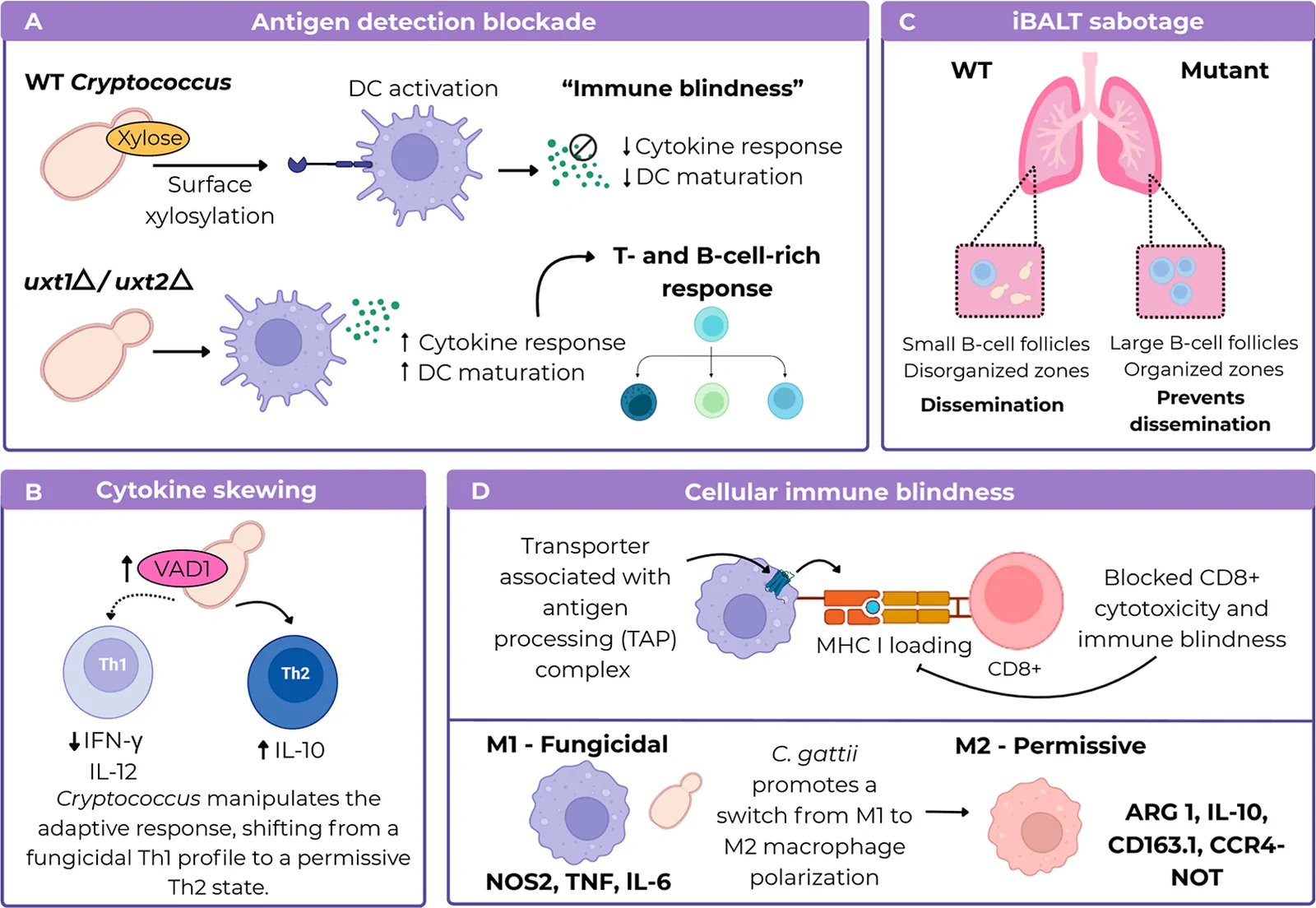
Multilevel interference of *Cryptococcus* with the adaptive immune response. (A) Surface xylosylation in wild-type strains impairs dendritic cell (DC) activation and maturation, reducing cytokine production and promoting a state of “immune blindness,” whereas uxt1Δ/uxt2Δmutants lacking proper xylosylation induce stronger DC activation and favor a protective T- and B-cell-rich response. (B) *C. neoformans* promotes cytokine skewing through VAD1, suppressing Th1-associated mediators, such as IFN-γ and IL-12, and favoring a permissive Th2 profile enriched in IL-10. (C) In the lung, capsular xylosylation disrupts the structural organization of inducible bronchus-associated lymphoid tissue (iBALT), generating small and disorganized B-cell follicles that permit dissemination, whereas mutant strains induce larger and more organized follicles that restrict fungal spread. (D) Highly virulent *C. gattii* strains suppress cellular immunity by downregulating antigen processing and presentation pathways, including the transporter associated with antigen processing (TAP) complex and MHC class I loading, thereby impairing CD8+ T-cell recognition. In parallel, these strains inhibit fungicidal M1 macrophage polarization and promote transition to a permissive M2 phenotype, favoring fungal persistence and immune evasion. Illustration created by the authors using Biorender (https://www.biorender.com/) and Canva (https://www.canva.com/). Graphical elements were used under valid publication licenses in accordance with the respective platform license agreements. Therefore, no additional permission from third-party copyright holders was required.

### 3 Perspectives

Despite significant advances in understanding the immune evasion mechanisms of *Cryptococcus* spp., several gaps still limit a more integrated view of the fungus’s pathogenesis and adaptation in the host. Much of the currently available knowledge has been built upon the characterization of classic virulence factors, such as the polysaccharide capsule, melanin, and cell wall remodeling. However, more recent evidence indicates that immune evasion does not depend on a single determinant, but rather on a dynamic network of structural, metabolic, and regulatory responses, capable of varying according to the infectious niche, the time of infection, and the host’s immune status.

In this context, one of the main challenges is understanding how these mechanisms operate in a coordinated manner throughout the infection. For example, it is not yet fully understood how alterations in fungal surface architecture are linked to intracellular metabolic changes, adaptation to oxidative stress, intraphagocytic survival, and dissemination to the CNS. Similarly, the temporal understanding of these events remains limited, since many studies evaluate evasion factors in isolation, without considering the progression of the fungal response from the initial stages of recognition to persistence or tissue dissemination.

Another important gap concerns the heterogeneity between species, lineages, and experimental contexts. Although *C. neoformans* and *C. gattii* share several immune escape strategies, evidence suggests that these species complexes may differ in the intensity, regulation, and biological impact of certain mechanisms. Thus, overgeneralizations should still be avoided, especially when data are derived from few isolates or specific experimental models. More robust comparative studies, including multiomics approaches, functional genetics, and more physiologically representative infection models, will be fundamental to more precisely defining which mechanisms are conserved and which are species-specific.

Furthermore, distinguishing associations from causality remains a central issue. In many cases, proteins, regulatory pathways, or phenotypic alterations are linked to immune evasion based on experimental correlations, but without sufficient functional validation to demonstrate their direct role in host interaction. In this sense, the integration of proteomics, transcriptomics, metabolomics, and phenotypic assays represents a promising strategy to transform partial mechanistic descriptions into more consistent biological models.

From a translational perspective, deepening the understanding of these processes could contribute to the identification of new biomarkers of disease progression and therapeutic targets capable of compromising fungal adaptation in the host. Molecules involved in PAMP masking, capsular organization, stress response, and intracellular survival stand out as particularly relevant candidates, especially given the existing therapeutic limitations for cryptococcosis. Therefore, future investigations should prioritize not only the description of virulence factors, but also the elucidation of how these elements functionally converge to sustain persistence, dissemination, and failure of immune control during cryptococcal infection.

## 4 Conclusion

Cryptococcal pathogenesis is a dynamic, multifactorial process requiring the coordinated integration of capsule modulation, PAMP masking, and metabolic adaptation to subvert host immunity and facilitate neurotropism. Although these individual mechanisms are well-described, their temporal orchestration and species-specific nuances remain significant knowledge gaps. Addressing this public health threat demands a synergistic approach, coupling functional genetics and infection models with high-resolution omics, to elucidate these complexities and accelerate the development of novel diagnostic and therapeutic strategies.
